# Bifurcation analysis and soliton solutions to the doubly dispersive equation in elastic inhomogeneous Murnaghan’s rod

**DOI:** 10.1038/s41598-024-62113-z

**Published:** 2024-05-19

**Authors:** S. M. Rayhanul Islam

**Affiliations:** Department of Mathematics, Pabna University of Science and Technology, Pabna, 6600 Bangladesh

**Keywords:** Doubly dispersive equation, Modified Khater method, Bifurcation analysis, Wave solutions, and equilibrium points, Mathematics and computing, Physics

## Abstract

The doubly dispersive (DD) equation finds extensive utility across scientific and engineering domains. It stands as a significant nonlinear physical model elucidating nonlinear wave propagation within the elastic inhomogeneous Murnaghan’s rod (EIMR). With this in mind, we have focused on the integration of the DD model and the modified Khater (MK) method. Through the wave transformation, this model is effectively converted into an ordinary differential equation. In this paper, the goal of our work is to explore new wave solutions to the DD model by using the MK scheme. These solutions provide extremely helpful insights into the operation of the system. The three-dimensional (3D) plot and two-dimensional (2D) combined plot via the impacts of the parameters are provided for various parameters in this manuscript. We also discussed the dynamical properties of the model, which are accomplished through the bifurcation analysis, and also found the Hamiltonian function. This research makes a substantial contribution to the area by increasing our understanding of wave solutions in the DD, introducing novel investigation tools, and carrying out an in-depth investigation of the bifurcation and stability aspects of the system. As a direct result of this research, novel openings have been uncovered for further investigation and application in the various disciplines of science and engineering.

## Introduction

The study of nonlinear evolution equations (NLEEs) has therefore become an exciting and pivotal topic in contemporary research in the last three decades. NLEEs have become a focal point of research due to their significance in understanding nonlinear, tangible phenomena. These equations serve as vital tools for analyzing and describing a wide range of nonlinear phenomena encountered in various fields, including nonlinear optics^1,2^, optical fibres^3,4^, communication systems^5^, fluid dynamics^6–9^, biology^10^, plasma physics^11^, and other scientific disciplines. It is important to look for traveling wave solutions and solitons because they help us understand how complex nonlinear properties work. By employing NLEEs, researchers can unravel the intricate dynamics and physical properties underlying these phenomena.

Understanding wave solutions of NLEEs is crucial for grasping the underlying physical mechanisms of the natural phenomena that NLEEs represent. These solutions offer significant insights into the structural characteristics of NLEEs, which are extensively employed in optical fibers, plasma physics, mathematical physics, and engineering applications. Various wave solutions, such as lump waves, dark waves, periodic waves, bright waves, solitary waves, and soliton waves, elucidate the phenomena modelled by NLEEs. In recent decades, soliton and solitary wave solutions have been extensively investigated by researchers across various nonlinear scientific fields. As a result, numerous mathematicians, physicists, and engineers have developed several reliable models to carry out progressive wave approximations for these nonlinear processes and have succeeded in identifying solutions using a variety of analytical and numerical methods. Among many techniques, some efficient and powerful schemes are the new Kudryashov^12^, the generalized projective Riccati equations^13^, the extended simple equation^14^, the Bernoulli sub-ode^15^, the ($w/g$)-expansion^16^, the unified and improved F-expansion^17^, the Galarkin^18,19^, the Khater II^20^, the He’s variational iteration^21^, the extended Khater^22^, the direct algebraic^23,24^, the modified Khater^25^, the generalized rational^26^, the modified direct algebraic^27^, the novel generalized Kudryashov^28^, the extended simplest equation^29^, the generalized exponential function^30^, the Bernoulli sub-equation^31^, and various other methods. Due to this study, considerably more attention has been given to solutions, structures, interactions, and other properties of methods, and various significant results have been successfully obtained.

Solitons exhibiting the capability to either divide or merge when they interact are termed bifurcation solitons. The significance of a soliton lies in its integrability, denoting the soliton's ability to be integrated within the nonlinear equation even when faced with initial local disturbances. This results in a solution characterized by a collection of solitons and linear dispersive waves. A notable nonlinear physical model that describes the propagation of nonlinear waves in elastic inhomogeneous circular cylinders, often known as Murnaghan's rods, is the DD model^32^. Understanding wave propagation in nonlinear elastic substances is greatly aided by this study. Seismology, acoustics, introscopy, analysis of unexpected destruction, long-distance energy transmission, vibro-impact treatments of hard materials, and non-destructive testing methods, particularly for pipelines, are just a few of the many domains in which this adaptable equation has found use. Additionally, it helps in understanding the internal structure and physical characteristics of different solids, including brass, steel, glass, and polymers, as well as describing phenomena like shock waves, tsunamis, and solitons. This model has been useful in analysing the propagation of strain waves in EIMR when dealing with elastic inhomogeneous media. In this investigation, we employ the elegant auxiliary equation approach to explore novel and precise wave solutions for the DD model. This particular model emerges from nonlinear two-directional long-wave models utilized in describing longitudinal waves within nonlinear dynamic elasticity. In this paper, we investigate the wave solutions to the DD model, which is derived from nonlinear two-directional long-wave models for longitudinal waves in nonlinear dynamic elasticity^33–37^ given by,

1.1 $${u}_{tt}-{\left(\frac{1}{\rho }{\left(Qu\right)}_{x}\right)}_{x}=\frac{\varepsilon }{2}{\left(\frac{1}{\rho }{\left(q\eta {u}^{2}+\rho \chi {u}_{tt}-{\left(a\gamma {\chi }^{2}{u}_{x}\right)}_{x}\right)}_{x}\right)}_{x},$$

where $u(x,t)$ denotes the strain wave function, $\rho$ denotes the density, $\varepsilon$ denotes the small parameters, $q=\frac{B}{Q}$ and $a=\frac{M}{Q}$ denotes the scale factors, and $\gamma$ denotes the poisson parameter. Various formulations of the DD model have been developed, tailored to the specific assumptions about the medium involved. For instance, in the presence of isotropic media or weak dispersion, these equations can be simplified. Among the research conducted, investigations have focused on the EIMR, employing generalized DDs. For instance, Cattani et al.^33^ have explored the wave solutionsof the DD model by using the extended sinh-Gordon equation expansion (EsGEE) and the modified exp(− φ(ζ))-expansion function (MEEF) methods. Dusunceli et al.^34^ have inspected the wave solutions of the DD model through the improved Bernoulli sub-equation function (IBsEF) method. Ahmed et al.^35^ have investigated the DD model for finding wave solutions through the improved modified extended tanh-function technique. Using the extended Kudryashov and Bernoulli-Riccati approaches, obtaining wave solutions of the DD model by Ozisik et al.^36^. Alquran and Al-Smadi^37^ recently investigated the DD model and its bidirectional wave solutions using modified rational sine–cosine (MRSC) and sinh-cosh functions (SCF), along with unified methods. Their work focused on exploring various solutions, particularly single-wave propagation patterns derived from the proposed model. Alharthi et al.^38^ have examined wave solutions of the DD model using the modified generalized exponential rational function method and the Jacobi elliptical finder method. Rehman et al.^39^ investigated wave solutions of the DD model, which characterizes nonlinear wave propagation in the EIMR, employing the Sardar sub-equation method. Younas et al.^40^ developed exact solutions for the DD model utilizing the new extended direct algebraic and generalized Kudryashov methods. Rathinavel et al.^41^ explored wave solutions of the DD model for wave propagation in a nonlinear EIMR, employing the $F$-expansion technique. Abourabia and Eldreeny^42^ inspected wave solutions to the DD model using the commutative hyper-complex algebraic scheme. Asjad et al.^43^ found wave solutions to the DD model via the direct algebraic extended technique. Yel^44^ determined the traveling wave solutions of the DD model in nonlinear dynamic elasticity through the sine–Gordon expansion scheme. Ibrahim et al.^45^ have examined the optical solitons for the DD using the Sardar sub-equation, which explains the flow of shallow water in a small-amplitude surface system. Separately, Eremeyev and Kolpakov^46^ employed a numerical approach to study nonlinear wave propagation in EIMR, demonstrating successful prediction of solitary wave formation and comprehensive property analysis. Additionally, Eremeyev et al.^47^ analyzed harmonic wave propagation in EIMR using the same model, deriving analytical solutions for dispersion relations and investigating material parameter influences on wave propagation.

As we can see, many methods have been discovered to serve NLEEs to obtain exact traveling wave solutions. Among them, Khater method is an analytical method to obtain solitary wave solutions of the NLEEs through these techniques^48^ in 2017. A few months letter, Bibi et al.^49^ have considered this method and applied to the nonlinear Sharma Tasso-Olever equation for exploring exact solutions. In 2018, Khater et al.^50^ have inspected the wave solutionsto the higher order nonlinear Sasa-Satsuma equation in mono mode fibers through the new auxiliary equation method, but the potential scholar invalidated some solutions of this method, which have been discussed in Refs.^51–53^. At the same time, El-Ganaini and Zayed gave us the correct form some of the solutions in Ref.^54^. After that, Khater introduced the modified Khater method, which is extension of the Khater method. It is applied to the Schwarzian Korteweg-de-Vries equation and (2 + 1)-Ablowitz-Kaup-Newell-Segur equation to obtain exact solutions^55,56^. However, we have considered modified Khater method in this paper and apply it to the model described in Section “Mathematical analysis”.

It is evident from an inspection of prior works by different researchers on the DD model that the MK techniques have not been previously employed, nor have wave solutionsbeen derived using this approach, and also did not discuss the impact of the parameters. Furthermore, none of these previous authors conducted the bifurcation analysis and demonstrated paths to stable solutions for the wave variable, which none of the previous authors discussed. This observation underscores the gap in the existing research literature that our study aims to address.

The main goal of this study is to generate wave solutions for the DD model using the MK scheme and also examining the influence of parameters. Additionally, we will also clarify the characteristics of the soliton pulse, offering both graphical and physical explanations within the context of the DD model. We discussed the bifurcation analysis of the model through the planar dynamical system. The Hamiltonian function is found and also drawn the phase portrait to identify the nature of the obtained solutions.

The rest of this paper is designed as follows: we have done the mathematical analysis in Sect. “Mathematical analysis” including applying the MK scheme to the DD model, and comparison between our solutions and previous literatures in the same section. The graphical and physical interpretation some solutions of the DD model and the implications of parameters are also discussed in Sect. “Graphical and physical explanations some of the solutions”. The bifurcation analysis of the model is constructed in Sect. “Bifurcation analysis of the DD model”. Finally, we offered a comprehensive conclusion to summarize our findings in Sect. "Conclusion".

## Mathematical analysis

This section introduces the MK method applied to the DD model, leading to the establishment of comprehensive wave solutions. By delving into the abundant wave solutions of the DD model, we aim to elucidate their pivotal role in modern science and engineering across various wave phenomena. Now, through the wave transformation as,

2.1 $$u\left(x,t\right)=\phi \left(\xi \right)\,\,\mathrm{ and }\,\,\xi =x-\omega t.$$

Now, switching Eq. (2.1) into Eq. (1.1) to obtain ODE (more details in Refs.^33–37^), yields,

2.2 $$2\left(Q-{\omega }^{2}\rho \right)\phi +q\eta \varepsilon {\phi }^{2}+\varepsilon \chi \left({\omega }^{2}\rho -\gamma a\right){\phi }^{{\prime}{\prime}}=0.$$

The linear wave transformation features a scalar $\omega$, which signifies the wave speed. As indicated in Eq. (2.2), the wave speed is represented by ${\omega }^{2}$. Upon scrutinising the solution of Eq. (2.2), it becomes evident that two distinct values of $\omega$ consistently coexist. As a result, the propagation of the DD model manifests as symmetric bi-directional waves in motion^37^.

Now, balancing the term ${\phi }^{{\prime}{\prime}}$ and ${\phi }^{2}$ in Eq. (2.2), gives $N=2$. The general solution takes the form

2.3 $$\phi \left(\xi \right)={c}_{0}+{c}_{1}{d}^{f(\xi )}+{c}_{2}{d}^{2f(\xi )},$$

where ${c}_{0},{c}_{1}$ and ${c}_{2}$ are constants but ${c}_{2}\ne 0$ and the function $f\left(\xi \right)$ satisfies the first order auxiliary equation ${f}^{\prime}\left(\xi \right)=\frac{1}{{\text{ln}}(d)}\left\{\lambda {d}^{-f(\xi )}+\mu +\sigma {d}^{f(\xi )}\right\}$. Relieving Eq. (2.3) into the Eq. (2.2), we get the algebraic equations and solve them, yields the solution sets:

2.4 $$\mathrm{Set \,\,one}:\theta =\pm \sqrt{\frac{4a\gamma \lambda \sigma \varepsilon \chi -a\gamma {\mu }^{2}\varepsilon \chi -2Q}{4\lambda \rho \sigma \varepsilon \chi -{\mu }^{2}\rho \varepsilon \chi -2\rho }}, {c}_{0}=-\frac{2\chi \left(2a\gamma \lambda \sigma +a\gamma {\mu }^{2}-2Q\lambda \sigma -Q{\mu }^{2}\right)}{\left(4\lambda \sigma \varepsilon \chi -{\mu }^{2}\varepsilon \chi -2\right)\eta q}, {c}_{1}=-\frac{12\mu \sigma \chi \left(a\gamma -Q\right)}{\left(4\lambda \sigma \varepsilon \chi -{\mu }^{2}\varepsilon \chi -2\right)\eta q}, {c}_{2}=-\frac{12{\sigma }^{2}\chi \left(a\gamma -Q\right)}{\left(4\lambda \sigma \varepsilon \chi -{\mu }^{2}\varepsilon \chi -2\right)\eta q}.$$

2.5 $$\mathrm{Set \,\,two}:\theta =\pm \sqrt{\frac{4a\gamma \lambda \sigma \varepsilon \chi -a\gamma {\mu }^{2}\varepsilon \chi +2Q}{4\lambda \rho \sigma \varepsilon \chi -{\mu }^{2}\rho \varepsilon \chi +2\rho }, }{c}_{0}=\frac{12\lambda \chi \sigma \left(a\gamma -Q\right)}{\left(4\lambda \sigma \varepsilon \chi -{\mu }^{2}\varepsilon \chi +2\right)\eta q}, {c}_{1}=\frac{12\mu \sigma \chi \left(a\gamma -Q\right)}{\left(4\lambda \sigma \varepsilon \chi -{\mu }^{2}\varepsilon \chi +2\right)\eta q}, {c}_{2}=\frac{12{\sigma }^{2}\chi \left(a\gamma -Q\right)}{\left(4\lambda \sigma \varepsilon \chi -{\mu }^{2}\varepsilon \chi +2\right)\eta q}.$$

When incorporating the provided estimates from (2.4) into the equation mentioned in (2.3), the resultant outcome is as follows:

When $${\mu }^{2}-4\lambda \sigma <0$$ and $$\sigma \ne 0,$$

$${u}_{\mathrm{1,2}}\left(x,t\right)=\frac{3\chi \left(Q-a\gamma \right)\left({\mu }^{2}-4\lambda \sigma \right)}{q\eta \left(2+\varepsilon \chi \left({\mu }^{2}-4\lambda \sigma \right)\right)}\left({tan}^{2}\left(\frac{\sqrt{4\lambda \sigma -{\mu }^{2}}}{2}\left(x\pm \sqrt{\frac{4a\gamma \lambda \sigma \varepsilon \chi -a\gamma {\mu }^{2}\varepsilon \chi -2Q}{4\lambda \rho \sigma \varepsilon \chi -{\mu }^{2}\rho \varepsilon \chi -2\rho }} t\right)\right)+\frac{1}{3}\right),$$

and

$${u}_{\mathrm{3,4}}\left(x,t\right)=\frac{3\chi \left(Q-a\gamma \right)\left({\mu }^{2}-4\lambda \sigma \right)}{q\eta \left(2+\varepsilon \chi \left({\mu }^{2}-4\lambda \sigma \right)\right)}\left({cot}^{2}\left(\frac{\sqrt{4\lambda \sigma -{\mu }^{2}}}{2}\left(x\pm \sqrt{\frac{4a\gamma \lambda \sigma \varepsilon \chi -a\gamma {\mu }^{2}\varepsilon \chi -2Q}{4\lambda \rho \sigma \varepsilon \chi -{\mu }^{2}\rho \varepsilon \chi -2\rho }} t\right)\right)+\frac{1}{3}\right).$$

When $${\mu }^{2}-4\lambda \sigma >0$$ and $$\sigma \ne 0,$$

$${u}_{\mathrm{5,6}}\left(x,t\right)=-\frac{3\chi \left(Q-a\gamma \right)\left({\mu }^{2}-4\lambda \sigma \right)}{q\eta \left(2+\varepsilon \chi \left({\mu }^{2}-4\lambda \sigma \right)\right)}\left({tanh}^{2}\left(\frac{\sqrt{{\mu }^{2}-4\lambda \sigma }}{2}\left(x\pm \sqrt{\frac{4a\gamma \lambda \sigma \varepsilon \chi -a\gamma {\mu }^{2}\varepsilon \chi -2Q}{4\lambda \rho \sigma \varepsilon \chi -{\mu }^{2}\rho \varepsilon \chi -2\rho }} t\right)\right)-\frac{1}{3}\right),$$

and

$${u}_{\mathrm{7,8}}\left(x,t\right)=-\frac{3\chi \left(Q-a\gamma \right)\left({\mu }^{2}-4\lambda \sigma \right)}{q\eta \left(2+\varepsilon \chi \left({\mu }^{2}-4\lambda \sigma \right)\right)}\left({coth}^{2}\left(\frac{\sqrt{{\mu }^{2}-4\lambda \sigma }}{2}\left(x\pm \sqrt{\frac{4a\gamma \lambda \sigma \varepsilon \chi -a\gamma {\mu }^{2}\varepsilon \chi -2Q}{4\lambda \rho \sigma \varepsilon \chi -{\mu }^{2}\rho \varepsilon \chi -2\rho }} t\right)\right)-\frac{1}{3}\right).$$

When $${\mu }^{2}+4{\lambda }^{2}<0,\sigma \ne 0$$ and $$\sigma =-\lambda ,$$

$${u}_{\mathrm{9,10}}\left(x,t\right)=\frac{3\chi \left(Q-a\gamma \right)\left({\mu }^{2}+4{\lambda }^{2}\right)}{q\eta \left(2+\varepsilon \chi \left({\mu }^{2}+4{\lambda }^{2}\right)\right)}\left({tan}^{2}\left(\frac{\sqrt{-{\mu }^{2}-4{\lambda }^{2}}}{2}\left(x\pm \sqrt{\frac{4a\gamma \lambda \sigma \varepsilon \chi -a\gamma {\mu }^{2}\varepsilon \chi -2Q}{4\lambda \rho \sigma \varepsilon \chi -{\mu }^{2}\rho \varepsilon \chi -2\rho }} t\right)\right)+\frac{1}{3}\right),$$

and

$${u}_{\mathrm{11,12}}\left(x,t\right)=\frac{3\chi \left(Q-a\gamma \right)\left({\mu }^{2}+4{\lambda }^{2}\right)}{q\eta \left(2+\varepsilon \chi \left({\mu }^{2}+4{\lambda }^{2}\right)\right)}\left({cot}^{2}\left(\frac{\sqrt{-{\mu }^{2}-4{\lambda }^{2}}}{2}\left(x\pm \sqrt{\frac{4a\gamma \lambda \sigma \varepsilon \chi -a\gamma {\mu }^{2}\varepsilon \chi -2Q}{4\lambda \rho \sigma \varepsilon \chi -{\mu }^{2}\rho \varepsilon \chi -2\rho }} t\right)\right)+\frac{1}{3}\right),$$

When $${\mu }^{2}+4{\lambda }^{2}>0,\sigma \ne 0$$ and $$\sigma =-\lambda ,$$

$${u}_{\mathrm{13,14}}\left(x,t\right)=-\frac{3\chi \left(Q-a\gamma \right)\left({\mu }^{2}+4{\lambda }^{2}\right)}{q\eta \left(2+\varepsilon \chi \left({\mu }^{2}+4{\lambda }^{2}\right)\right)}\left({tanh}^{2}\left(\frac{\sqrt{{\mu }^{2}+4{\lambda }^{2}}}{2}\left(x\pm \sqrt{\frac{4a\gamma \lambda \sigma \varepsilon \chi -a\gamma {\mu }^{2}\varepsilon \chi -2Q}{4\lambda \rho \sigma \varepsilon \chi -{\mu }^{2}\rho \varepsilon \chi -2\rho }} t\right)\right)-\frac{1}{3}\right),$$

and

$${u}_{\mathrm{15,16}}\left(x,t\right)=-\frac{3\chi \left(Q-a\gamma \right)\left({\mu }^{2}+4{\lambda }^{2}\right)}{q\eta \left(2+\varepsilon \chi \left({\mu }^{2}+4{\lambda }^{2}\right)\right)}\left({coth}^{2}\left(\frac{\sqrt{{\mu }^{2}+4{\lambda }^{2}}}{2}\left(x\pm \sqrt{\frac{4a\gamma \lambda \sigma \varepsilon \chi -a\gamma {\mu }^{2}\varepsilon \chi -2Q}{4\lambda \rho \sigma \varepsilon \chi -{\mu }^{2}\rho \varepsilon \chi -2\rho }} t\right)\right)-\frac{1}{3}\right).$$

When $${\mu }^{2}-4{\lambda }^{2}<0$$ and $$\sigma =\lambda ,$$

$${u}_{\mathrm{17,18}}\left(x,t\right)=\frac{3\chi \left(Q-a\gamma \right)\left({\mu }^{2}-4{\lambda }^{2}\right)}{q\eta \left(2+\varepsilon \chi \left({\mu }^{2}-4{\lambda }^{2}\right)\right)}\left({tan}^{2}\left(\frac{\sqrt{4{\lambda }^{2}-{\mu }^{2}}}{2}\left(x\pm \sqrt{\frac{4a\gamma \lambda \sigma \varepsilon \chi -a\gamma {\mu }^{2}\varepsilon \chi -2Q}{4\lambda \rho \sigma \varepsilon \chi -{\mu }^{2}\rho \varepsilon \chi -2\rho }} t\right)\right)+\frac{1}{3}\right),$$

and

$${u}_{\mathrm{19,20}}\left(x,t\right)=\frac{3\chi \left(Q-a\gamma \right)\left({\mu }^{2}-4{\lambda }^{2}\right)}{q\eta \left(2+\varepsilon \chi \left({\mu }^{2}-4{\lambda }^{2}\right)\right)}\left({cot}^{2}\left(\frac{\sqrt{-{\mu }^{2}-4{\lambda }^{2}}}{2}\left(x\pm \sqrt{\frac{4a\gamma \lambda \sigma \varepsilon \chi -a\gamma {\mu }^{2}\varepsilon \chi -2Q}{4\lambda \rho \sigma \varepsilon \chi -{\mu }^{2}\rho \varepsilon \chi -2\rho }} t\right)\right)+\frac{1}{3}\right).$$

When $${\mu }^{2}-4{\lambda }^{2}>0$$ and $$\sigma =\lambda ,$$

$${u}_{\mathrm{21,22}}\left(x,t\right)=-\frac{3\chi \left(Q-a\gamma \right)\left({\mu }^{2}-4{\lambda }^{2}\right)}{q\eta \left(2+\varepsilon \chi \left({\mu }^{2}-4{\lambda }^{2}\right)\right)}\left({tanh}^{2}\left(\frac{\sqrt{{\mu }^{2}-4{\lambda }^{2}}}{2}\left(x\pm \sqrt{\frac{4a\gamma \lambda \sigma \varepsilon \chi -a\gamma {\mu }^{2}\varepsilon \chi -2Q}{4\lambda \rho \sigma \varepsilon \chi -{\mu }^{2}\rho \varepsilon \chi -2\rho }} t\right)\right)-\frac{1}{3}\right),$$

and

$${u}_{\mathrm{23,24}}\left(x,t\right)=-\frac{3\chi \left(Q-a\gamma \right)\left({\mu }^{2}-4{\lambda }^{2}\right)}{q\eta \left(2+\varepsilon \chi \left({\mu }^{2}-4{\lambda }^{2}\right)\right)}\left({coth}^{2}\left(\frac{\sqrt{{\mu }^{2}-4{\lambda }^{2}}}{2}\left(x\pm \sqrt{\frac{4a\gamma \lambda \sigma \varepsilon \chi -a\gamma {\mu }^{2}\varepsilon \chi -2Q}{4\lambda \rho \sigma \varepsilon \chi -{\mu }^{2}\rho \varepsilon \chi -2\rho }} t\right)\right)-\frac{1}{3}\right).$$

When $${\mu }^{2}=4\lambda \sigma ,$$

$${u}_{\mathrm{25,26}}\left(x,t\right)=-\frac{6\chi \left(Q-a\gamma \right)}{q\eta {\left(x\pm \sqrt{\frac{4a\gamma \lambda \sigma \varepsilon \chi -a\gamma {\mu }^{2}\varepsilon \chi -2Q}{4\lambda \rho \sigma \varepsilon \chi -{\mu }^{2}\rho \varepsilon \chi -2\rho }} t\right)}^{2}}.$$

When $$\lambda \sigma <0,\mu =0$$ and $$\sigma \ne 0,$$

$${u}_{\mathrm{27,28}}\left(x,t\right)=-\frac{4\chi \lambda \sigma \left(Q-a\gamma \right)}{q\eta \left(2\varepsilon \sigma \lambda \chi -1\right)}\times \frac{\left({cosh}^{2}\left(\sqrt{-\lambda \sigma } \left(x\pm \sqrt{\frac{4a\gamma \lambda \sigma \varepsilon \chi -a\gamma {\mu }^{2}\varepsilon \chi -2Q}{4\lambda \rho \sigma \varepsilon \chi -{\mu }^{2}\rho \varepsilon \chi -2\rho }} t\right)\right)-\frac{3}{2}\right)}{{cosh}^{2}\left(\sqrt{-\lambda \sigma } \left(x\pm \sqrt{\frac{4a\gamma \lambda \sigma \varepsilon \chi -a\gamma {\mu }^{2}\varepsilon \chi -2Q}{4\lambda \rho \sigma \varepsilon \chi -{\mu }^{2}\rho \varepsilon \chi -2\rho }} t\right)\right)},$$

and

$${u}_{\mathrm{29,30}}\left(x,t\right)=-\frac{4\chi \lambda \sigma \left(Q-a\gamma \right)}{q\eta \left(2\varepsilon \sigma \lambda \chi -1\right)}\times \frac{\left({cosh}^{2}\left(\sqrt{-\lambda \sigma } \left(x\pm \sqrt{\frac{4a\gamma \lambda \sigma \varepsilon \chi -a\gamma {\mu }^{2}\varepsilon \chi -2Q}{4\lambda \rho \sigma \varepsilon \chi -{\mu }^{2}\rho \varepsilon \chi -2\rho }} t\right)\right)+\frac{1}{2}\right)}{{sinh}^{2}\left(\sqrt{-\lambda \sigma } \left(x\pm \sqrt{\frac{4a\gamma \lambda \sigma \varepsilon \chi -a\gamma {\mu }^{2}\varepsilon \chi -2Q}{4\lambda \rho \sigma \varepsilon \chi -{\mu }^{2}\rho \varepsilon \chi -2\rho }} t\right)\right)}.$$

When $$\mu =0$$ and $$\lambda =-\sigma ,$$

$${u}_{\mathrm{31,32}}\left(x,t\right)=-\frac{4\chi {\sigma }^{2}\left(Q-a\gamma \right)}{q\eta \left(2\varepsilon {\sigma }^{2}\chi +1\right)}\times \frac{{e}^{-4\sigma \xi }+4{e}^{-2\sigma \xi }+1}{{\left({e}^{-2\sigma \xi }-1\right)}^{2}}, \xi =x\pm \sqrt{\frac{4a\gamma \lambda \sigma \varepsilon \chi -a\gamma {\mu }^{2}\varepsilon \chi -2Q}{4\lambda \rho \sigma \varepsilon \chi -{\mu }^{2}\rho \varepsilon \chi -2\rho }} t.$$

When $$\mu =\sigma =K$$ and $$\lambda =0,$$

$${u}_{\mathrm{33,34}}\left(x,t\right)=-\frac{2{K}^{2}\chi \left(Q-a\gamma \right)}{\eta q\left(\varepsilon \chi {K}^{2}+2\right)}\times \frac{{e}^{2K\xi }+4{e}^{K\xi }+1}{{\left({e}^{K\xi }-1\right)}^{2}}, \xi =x\pm \sqrt{\frac{4a\gamma \lambda \sigma \varepsilon \chi -a\gamma {\mu }^{2}\varepsilon \chi -2Q}{4\lambda \rho \sigma \varepsilon \chi -{\mu }^{2}\rho \varepsilon \chi -2\rho }} t.$$

When $$\mu =(\lambda +\sigma ),$$

$${u}_{\mathrm{35,36}}\left(x,t\right)=-\frac{8\chi {\left(\sigma -\lambda \right)}^{2}\left(Q-a\gamma \right)}{\eta q\left(\varepsilon \chi {\lambda }^{2}-2\lambda \chi \varepsilon \sigma +{\sigma }^{2}\chi \varepsilon +2\right)}\times \frac{{\frac{1}{4}{\sigma }^{2}e}^{2\left(\lambda -\sigma \right)\xi }+\sigma {e}^{\left(\lambda -\sigma \right)\xi }+\frac{1}{4}}{{\left(\sigma {e}^{\left(\lambda -\sigma \right)\xi }-1\right)}^{2}}, \xi =x\pm \sqrt{\frac{4a\gamma \lambda \sigma \varepsilon \chi -a\gamma {\mu }^{2}\varepsilon \chi -2Q}{4\lambda \rho \sigma \varepsilon \chi -{\mu }^{2}\rho \varepsilon \chi -2\rho }} t.$$

When $$\mu =-(\lambda +\sigma ),$$

$${u}_{\mathrm{37,38}}\left(x,t\right)=-\frac{8\chi {\left(\sigma -\lambda \right)}^{2}\left(Q-a\gamma \right)}{\eta q\left(\varepsilon \chi {\lambda }^{2}-2\lambda \chi \varepsilon \sigma +{\sigma }^{2}\chi \varepsilon +2\right)}\times \frac{{\frac{1}{4}e}^{2\left(\lambda -\sigma \right)\xi }+\sigma {e}^{\left(\lambda -\sigma \right)\xi }+\frac{{\sigma }^{2}}{4}}{{\left({e}^{\left(\lambda -\sigma \right)\xi }-\sigma \right)}^{2}}, \xi =x\pm \sqrt{\frac{4a\gamma \lambda \sigma \varepsilon \chi -a\gamma {\mu }^{2}\varepsilon \chi -2Q}{4\lambda \rho \sigma \varepsilon \chi -{\mu }^{2}\rho \varepsilon \chi -2\rho }} t.$$

When $$\lambda =0$$,

$${u}_{\mathrm{39,40}}\left(x,t\right)=-\frac{2{\mu }^{2}\chi \left(Q-a\gamma \right)}{\eta q\left(\varepsilon \chi {\mu }^{2}+2\right)}\times \frac{\frac{1}{4}{\sigma }^{2}{e}^{2\mu \xi }+\sigma {e}^{\mu \xi }+\frac{1}{4}}{{\left(\sigma {e}^{\mu \xi }-1\right)}^{2}}, \xi =x\pm \sqrt{\frac{4a\gamma \lambda \sigma \varepsilon \chi -a\gamma {\mu }^{2}\varepsilon \chi -2Q}{4\lambda \rho \sigma \varepsilon \chi -{\mu }^{2}\rho \varepsilon \chi -2\rho }} t.$$

When $$\sigma =\mu =\lambda \ne 0,$$

$${u}_{\mathrm{41,42}}\left(x,t\right)=\frac{9{\lambda }^{2}\chi \left(Q-a\gamma \right)}{\eta q\left(3\varepsilon \chi {\lambda }^{2}-2\right)}\times \left({tan}^{2}\left(\frac{\sqrt{3}\lambda }{2}\left(x\pm \sqrt{\frac{4a\gamma \lambda \sigma \varepsilon \chi -a\gamma {\mu }^{2}\varepsilon \chi -2Q}{4\lambda \rho \sigma \varepsilon \chi -{\mu }^{2}\rho \varepsilon \chi -2\rho }} t\right)\right)+\frac{1}{3}\right).$$

When $$\lambda =\mu =0,$$

$${u}_{\mathrm{43,44}}\left(x,t\right)=-\frac{6\chi \left(Q-a\gamma \right)}{\eta q{\xi }^{2}}, \xi =x\pm \sqrt{\frac{4a\gamma \lambda \sigma \varepsilon \chi -a\gamma {\mu }^{2}\varepsilon \chi -2Q}{4\lambda \rho \sigma \varepsilon \chi -{\mu }^{2}\rho \varepsilon \chi -2\rho }} t.$$

When $$\sigma =\lambda$$ and $$\mu =0,$$

$${u}_{\mathrm{45,46}}\left(x,t\right)=-\frac{4{\lambda }^{2}\chi \left(Q-a\gamma \right)}{\eta q\left(2\varepsilon \chi {\lambda }^{2}-1\right)}\times {sec}^{2}\left(\lambda \left(x\pm \sqrt{\frac{4a\gamma \lambda \sigma \varepsilon \chi -a\gamma {\mu }^{2}\varepsilon \chi -2Q}{4\lambda \rho \sigma \varepsilon \chi -{\mu }^{2}\rho \varepsilon \chi -2\rho }} t\right)\right)\times \left({cos}^{2}\left(\lambda \left(x\pm \sqrt{\frac{4a\gamma \lambda \sigma \varepsilon \chi -a\gamma {\mu }^{2}\varepsilon \chi -2Q}{4\lambda \rho \sigma \varepsilon \chi -{\mu }^{2}\rho \varepsilon \chi -2\rho }} t\right)\right)-\frac{3}{2}\right).$$

Under certain conditions, namely $\lambda =\sigma =0$, $\lambda =\mu =K$ and $=0$, and $\sigma =0$, when the constants are substituted, constant solutions are obtained. However, these solutions are not presented here as they lack physical significance. The solution of the Eq. (1.1) with the mentioned method does not exist when $\mu =\sigma =0$.

When incorporating the provided estimates from (2.5) into the equation mentioned in (2.3), the resultant outcome is as follows:

When $${\mu }^{2}-4\lambda \sigma <0$$ and $$\sigma \ne 0,$$

$${u}_{\mathrm{47,48}}\left(x,t\right)=-\frac{3\chi \left(Q-a\gamma \right)\left({\mu }^{2}-4\lambda \sigma \right)}{q\eta \left(\varepsilon \chi \left({\mu }^{2}-4\lambda \sigma \right)-2\right)}\left({tan}^{2}\left(\frac{\sqrt{4\lambda \sigma -{\mu }^{2}}}{2}\left(x\pm \sqrt{\frac{4a\gamma \lambda \sigma \varepsilon \chi -a\gamma {\mu }^{2}\varepsilon \chi +2Q}{4\lambda \rho \sigma \varepsilon \chi -{\mu }^{2}\rho \varepsilon \chi +2\rho }} t\right)\right)+1\right),$$

and

$${u}_{\mathrm{49,50}}\left(x,t\right)=-\frac{3\chi \left(Q-a\gamma \right)\left({\mu }^{2}-4\lambda \sigma \right)}{q\eta \left(\varepsilon \chi \left({\mu }^{2}-4\lambda \sigma \right)-2\right)}\left({cot}^{2}\left(\frac{\sqrt{4\lambda \sigma -{\mu }^{2}}}{2}\left(x\pm \sqrt{\frac{4a\gamma \lambda \sigma \varepsilon \chi -a\gamma {\mu }^{2}\varepsilon \chi +2Q}{4\lambda \rho \sigma \varepsilon \chi -{\mu }^{2}\rho \varepsilon \chi +2\rho }} t\right)\right)+1\right).$$

When $${\mu }^{2}-4\lambda \sigma >0$$ and $$\sigma \ne 0,$$

$${u}_{\mathrm{51,52}}\left(x,t\right)=\frac{3\chi \left(Q-a\gamma \right)\left({\mu }^{2}-4\lambda \sigma \right)}{q\eta \left(\varepsilon \chi \left({\mu }^{2}-4\lambda \sigma \right)-2\right)}\left({tanh}^{2}\left(\frac{\sqrt{{\mu }^{2}-4\lambda \sigma }}{2}\left(x\pm \sqrt{\frac{4a\gamma \lambda \sigma \varepsilon \chi -a\gamma {\mu }^{2}\varepsilon \chi +2Q}{4\lambda \rho \sigma \varepsilon \chi -{\mu }^{2}\rho \varepsilon \chi +2\rho }} t\right)\right)-1\right),$$

and

$${u}_{\mathrm{53,54}}\left(x,t\right)=\frac{3\chi \left(Q-a\gamma \right)\left({\mu }^{2}-4\lambda \sigma \right)}{q\eta \left(\varepsilon \chi \left({\mu }^{2}-4\lambda \sigma \right)-2\right)}\left({coth}^{2}\left(\frac{\sqrt{{\mu }^{2}-4\lambda \sigma }}{2}\left(x\pm \sqrt{\frac{4a\gamma \lambda \sigma \varepsilon \chi -a\gamma {\mu }^{2}\varepsilon \chi +2Q}{4\lambda \rho \sigma \varepsilon \chi -{\mu }^{2}\rho \varepsilon \chi +2\rho }} t\right)\right)-1\right).$$

When $${\mu }^{2}+4{\lambda }^{2}<0,\sigma \ne 0$$ and $$\sigma =-\lambda ,$$

$${u}_{\mathrm{55,56}}\left(x,t\right)=-\frac{3\chi \left(Q-a\gamma \right)\left({\mu }^{2}+4{\lambda }^{2}\right)}{q\eta \left(\varepsilon \chi \left({\mu }^{2}+4{\lambda }^{2}\right)-2\right)}\left({tan}^{2}\left(\frac{\sqrt{-{\mu }^{2}-4{\lambda }^{2}}}{2}\left(x\pm \sqrt{\frac{4a\gamma \lambda \sigma \varepsilon \chi -a\gamma {\mu }^{2}\varepsilon \chi +2Q}{4\lambda \rho \sigma \varepsilon \chi -{\mu }^{2}\rho \varepsilon \chi +2\rho }} t\right)\right)+1\right),$$

and

$${u}_{\mathrm{57,58}}\left(x,t\right)=-\frac{3\chi \left(Q-a\gamma \right)\left({\mu }^{2}+4{\lambda }^{2}\right)}{q\eta \left(\varepsilon \chi \left({\mu }^{2}+4{\lambda }^{2}\right)-2\right)}\left({cot}^{2}\left(\frac{\sqrt{-{\mu }^{2}-4{\lambda }^{2}}}{2}\left(x\pm \sqrt{\frac{4a\gamma \lambda \sigma \varepsilon \chi -a\gamma {\mu }^{2}\varepsilon \chi +2Q}{4\lambda \rho \sigma \varepsilon \chi -{\mu }^{2}\rho \varepsilon \chi +2\rho }} t\right)\right)+1\right).$$

When $${\mu }^{2}+4{\lambda }^{2}>0,\sigma \ne 0$$ and $$\sigma =-\lambda ,$$

$${u}_{\mathrm{59,60}}\left(x,t\right)=\frac{3\chi \left(Q-a\gamma \right)\left({\mu }^{2}+4{\lambda }^{2}\right)}{q\eta \left(\varepsilon \chi \left({\mu }^{2}+4{\lambda }^{2}\right)-2\right)}\left({tanh}^{2}\left(\frac{\sqrt{{\mu }^{2}+4{\lambda }^{2}}}{2}\left(x\pm \sqrt{\frac{4a\gamma \lambda \sigma \varepsilon \chi -a\gamma {\mu }^{2}\varepsilon \chi +2Q}{4\lambda \rho \sigma \varepsilon \chi -{\mu }^{2}\rho \varepsilon \chi +2\rho }} t\right)\right)-1\right),$$

and

$${u}_{\mathrm{61,62}}\left(x,t\right)=\frac{3\chi \left(Q-a\gamma \right)\left({\mu }^{2}+4{\lambda }^{2}\right)}{q\eta \left(\varepsilon \chi \left({\mu }^{2}+4{\lambda }^{2}\right)-2\right)}\left({coth}^{2}\left(\frac{\sqrt{{\mu }^{2}+4{\lambda }^{2}}}{2}\left(x\pm \sqrt{\frac{4a\gamma \lambda \sigma \varepsilon \chi -a\gamma {\mu }^{2}\varepsilon \chi +2Q}{4\lambda \rho \sigma \varepsilon \chi -{\mu }^{2}\rho \varepsilon \chi +2\rho }} t\right)\right)-1\right).$$

When $${\mu }^{2}-4{\lambda }^{2}<0$$ and $$\sigma =\lambda ,$$

$${u}_{\mathrm{63,64}}\left(x,t\right)=-\frac{3\chi \left(Q-a\gamma \right)\left({\mu }^{2}-4{\lambda }^{2}\right)}{q\eta \left(\varepsilon \chi \left({\mu }^{2}-4{\lambda }^{2}\right)-2\right)}\left({tan}^{2}\left(\frac{\sqrt{4{\lambda }^{2}-{\mu }^{2}}}{2}\left(x\pm \sqrt{\frac{4a\gamma \lambda \sigma \varepsilon \chi -a\gamma {\mu }^{2}\varepsilon \chi +2Q}{4\lambda \rho \sigma \varepsilon \chi -{\mu }^{2}\rho \varepsilon \chi +2\rho }} t\right)\right)+1\right),$$

and

$${u}_{\mathrm{65,66}}\left(x,t\right)=-\frac{3\chi \left(Q-a\gamma \right)\left({\mu }^{2}-4{\lambda }^{2}\right)}{q\eta \left(\varepsilon \chi \left({\mu }^{2}-4{\lambda }^{2}\right)-2\right)}\left({cot}^{2}\left(\frac{\sqrt{-{\mu }^{2}-4{\lambda }^{2}}}{2}\left(x\pm \sqrt{\frac{4a\gamma \lambda \sigma \varepsilon \chi -a\gamma {\mu }^{2}\varepsilon \chi +2Q}{4\lambda \rho \sigma \varepsilon \chi -{\mu }^{2}\rho \varepsilon \chi +2\rho }} t\right)\right)+1\right).$$

When $${\mu }^{2}-4{\lambda }^{2}>0$$ and $$\sigma =\lambda ,$$

$${u}_{\mathrm{67,68}}\left(x,t\right)=\frac{3\chi \left(Q-a\gamma \right)\left({\mu }^{2}-4{\lambda }^{2}\right)}{q\eta \left(\varepsilon \chi \left({\mu }^{2}-4{\lambda }^{2}\right)-2\right)}\left({tanh}^{2}\left(\frac{\sqrt{{\mu }^{2}-4{\lambda }^{2}}}{2}\left(x\pm \sqrt{\frac{4a\gamma \lambda \sigma \varepsilon \chi -a\gamma {\mu }^{2}\varepsilon \chi +2Q}{4\lambda \rho \sigma \varepsilon \chi -{\mu }^{2}\rho \varepsilon \chi +2\rho }} t\right)\right)-1\right),$$

and

$${u}_{\mathrm{69,70}}\left(x,t\right)=\frac{3\chi \left(Q-a\gamma \right)\left({\mu }^{2}-4{\lambda }^{2}\right)}{q\eta \left(\varepsilon \chi \left({\mu }^{2}-4{\lambda }^{2}\right)-2\right)}\left({coth}^{2}\left(\frac{\sqrt{{\mu }^{2}-4{\lambda }^{2}}}{2}\left(x\pm \sqrt{\frac{4a\gamma \lambda \sigma \varepsilon \chi -a\gamma {\mu }^{2}\varepsilon \chi +2Q}{4\lambda \rho \sigma \varepsilon \chi -{\mu }^{2}\rho \varepsilon \chi +2\rho }} t\right)\right)-1\right).$$

When $${\mu }^{2}=4\lambda \sigma ,$$

$${u}_{\mathrm{71,72}}\left(x,t\right)=-\frac{6\chi \left(Q-a\gamma \right)}{q\eta {\left(x\pm \sqrt{\frac{4a\gamma \lambda \sigma \varepsilon \chi -a\gamma {\mu }^{2}\varepsilon \chi +2Q}{4\lambda \rho \sigma \varepsilon \chi -{\mu }^{2}\rho \varepsilon \chi +2\rho }} t\right)}^{2}}.$$

When $$\lambda \sigma <0,\mu =0$$ and $$\sigma \ne 0,$$

$${u}_{\mathrm{73,74}}\left(x,t\right)=-\frac{6\chi \lambda \sigma \left(Q-a\gamma \right)}{q\eta \left(2\varepsilon \sigma \lambda \chi +1\right)}\times {sech}^{2}\left(\sqrt{-\lambda \sigma } \left(x\pm \sqrt{\frac{4a\gamma \lambda \sigma \varepsilon \chi -a\gamma {\mu }^{2}\varepsilon \chi +2Q}{4\lambda \rho \sigma \varepsilon \chi -{\mu }^{2}\rho \varepsilon \chi +2\rho }} t\right)\right),$$

and

$${u}_{\mathrm{75,76}}\left(x,t\right)=\frac{6\chi \lambda \sigma \left(Q-a\gamma \right)}{q\eta \left(2\varepsilon \sigma \lambda \chi +1\right)}\times {cosech}^{2}\left(\sqrt{-\lambda \sigma } \left(x\pm \sqrt{\frac{4a\gamma \lambda \sigma \varepsilon \chi -a\gamma {\mu }^{2}\varepsilon \chi +2Q}{4\lambda \rho \sigma \varepsilon \chi -{\mu }^{2}\rho \varepsilon \chi +2\rho }} t\right)\right).$$

When $$\mu =0$$ and $$\lambda =-\sigma ,$$

$${u}_{\mathrm{77,78}}\left(x,t\right)=\frac{24\chi {\sigma }^{2}\left(Q-a\gamma \right)}{q\eta \left(2\varepsilon {\sigma }^{2}\chi -1\right)}\times \frac{{e}^{-2\sigma \xi }}{{\left({e}^{-2\sigma \xi }-1\right)}^{2}}, \xi =x\pm \sqrt{\frac{4a\gamma \lambda \sigma \varepsilon \chi -a\gamma {\mu }^{2}\varepsilon \chi +2Q}{4\lambda \rho \sigma \varepsilon \chi -{\mu }^{2}\rho \varepsilon \chi +2\rho }}t.$$

When $$\mu =\sigma =K$$ and $$\lambda =0,$$

$${u}_{\mathrm{79,80}}\left(x,t\right)=\frac{12{K}^{2}\chi \left(Q-a\gamma \right)}{\eta q\left(\varepsilon \chi {K}^{2}-2\right)}\times \frac{{e}^{K\xi }}{{\left({e}^{K\xi }-1\right)}^{2}}, \xi =x\pm \sqrt{\frac{4a\gamma \lambda \sigma \varepsilon \chi -a\gamma {\mu }^{2}\varepsilon \chi +2Q}{4\lambda \rho \sigma \varepsilon \chi -{\mu }^{2}\rho \varepsilon \chi +2\rho }}t.$$

When $$\mu =(\lambda +\sigma ),$$

$${u}_{\mathrm{81,82}}\left(x,t\right)=\frac{12\sigma \chi {\left(\sigma -\lambda \right)}^{2}\left(Q-a\gamma \right)}{\eta q\left(\varepsilon \chi {\lambda }^{2}-2\lambda \chi \varepsilon \sigma +{\sigma }^{2}\chi \varepsilon -2\right)}\times \frac{{e}^{\left(\lambda -\sigma \right)\xi }}{{\left(\sigma {e}^{\left(\lambda -\sigma \right)\xi }-1\right)}^{2}}, \xi =x\pm \sqrt{\frac{4a\gamma \lambda \sigma \varepsilon \chi -a\gamma {\mu }^{2}\varepsilon \chi +2Q}{4\lambda \rho \sigma \varepsilon \chi -{\mu }^{2}\rho \varepsilon \chi +2\rho }}t.$$

When $$\mu =-(\lambda +\sigma ),$$

$${u}_{\mathrm{83,84}}\left(x,t\right)=\frac{12\chi {\left(\sigma -\lambda \right)}^{2}\left(Q-a\gamma \right)}{\eta q\left(\varepsilon \chi {\lambda }^{2}-2\lambda \chi \varepsilon \sigma +{\sigma }^{2}\chi \varepsilon -2\right)}\times \frac{\sigma {e}^{\left(\lambda -\sigma \right)\xi }}{{\left({e}^{\left(\lambda -\sigma \right)\xi }-\sigma \right)}^{2}}, \xi =x\pm \sqrt{\frac{4a\gamma \lambda \sigma \varepsilon \chi -a\gamma {\mu }^{2}\varepsilon \chi +2Q}{4\lambda \rho \sigma \varepsilon \chi -{\mu }^{2}\rho \varepsilon \chi +2\rho }}t.$$

When $$\lambda =0$$,

$${u}_{\mathrm{85,86}}\left(x,t\right)=\frac{12{\mu }^{2}\sigma \chi \left(Q-a\gamma \right)}{\eta q\left(\varepsilon \chi {\mu }^{2}-2\right)}\times \frac{{e}^{\mu \xi }}{{\left(\sigma {e}^{\mu \xi }-1\right)}^{2}}, \xi =x\pm \sqrt{\frac{4a\gamma \lambda \sigma \varepsilon \chi -a\gamma {\mu }^{2}\varepsilon \chi +2Q}{4\lambda \rho \sigma \varepsilon \chi -{\mu }^{2}\rho \varepsilon \chi +2\rho }}t.$$

When $$\sigma =\mu =\lambda \ne 0,$$

$${u}_{\mathrm{87,88}}\left(x,t\right)=-\frac{9{\lambda }^{2}\chi \left(Q-a\gamma \right)}{\eta q\left(3\varepsilon \chi {\lambda }^{2}+2\right)}\times \left({tan}^{2}\left(\frac{\sqrt{3}\lambda }{2}\left(x\pm \sqrt{\frac{4a\gamma \lambda \sigma \varepsilon \chi -a\gamma {\mu }^{2}\varepsilon \chi +2Q}{4\lambda \rho \sigma \varepsilon \chi -{\mu }^{2}\rho \varepsilon \chi +2\rho }} t\right)\right)+1\right).$$

When $$\lambda =\mu =0,$$

$${u}_{\mathrm{89,90}}\left(x,t\right)=-\frac{6\chi \left(Q-a\gamma \right)}{\eta q{\xi }^{2}}, \xi =x\pm \sqrt{\frac{4a\gamma \lambda \sigma \varepsilon \chi -a\gamma {\mu }^{2}\varepsilon \chi +2Q}{4\lambda \rho \sigma \varepsilon \chi -{\mu }^{2}\rho \varepsilon \chi +2\rho }}t.$$

When $$\sigma =\lambda$$ and $$\mu =0,$$

$${u}_{\mathrm{91,92}}\left(x,t\right)=-\frac{6{\lambda }^{2}\chi \left(Q-a\gamma \right)}{\eta q\left(2\varepsilon \chi {\lambda }^{2}+1\right)}\times {sec}^{2}\left(\lambda \left(x\pm \sqrt{\frac{4a\gamma {\lambda }^{2}\varepsilon \chi +2Q}{4{\lambda }^{2}\rho \varepsilon \chi +2\rho }} t\right)\right).$$

The solution of the Eq. (1.1) with the mentioned method do not exists when $\mu =\sigma =0$, $\lambda =\sigma =0$, $\lambda =\mu =K$ and $\sigma =0$, and $\sigma =0$.

### Comparison

Alquran and Smadi^37^ have inspected ten wave solutions of the DD model using the MRSC and SCF, and unified approaches. Cattani et al.^33^ have constructed fifteen solutions of the DD model through the EsGEE and MEEF techniques. Dusunceli et al.^34^ have constructed ten solutions of the DD model by using the IBsEF technique. But, in our present paper, we employed the MK scheme and independently uncovered ninety-two solutions from the DD model. These solutions are expressed as the rational function solution, exponential function solution, trigonometric function solution and hyperbolic function solution. As a result, both methods have a common solution of the DD model, whose solutions does not shown in this manuscript. Finally, we can say that the solutions obtained in our research differ from those reported in Refs.^33,34,37^.

## Graphical and physical explanations some of the solutions

The DD model provides a variety of novel wave solutions expressed in exponential, trigonometric, and hyperbolic functions such as tanh, coth, sec, cos, tan, and cot, along with their combinations. These solutions encompass periodic-wave solutions, kink waves, combinations of kink and multi-solitons, periodic lumps, and periodic solitons. In the following section, we delve into the physical interpretations of these derived solutions, examine the effects of the parameters, conduct a bifurcation analysis of the model, and perform numerical simulations with various parameter values.

### Some 3D wave profiles of the attained solutions

**Figure 1:**
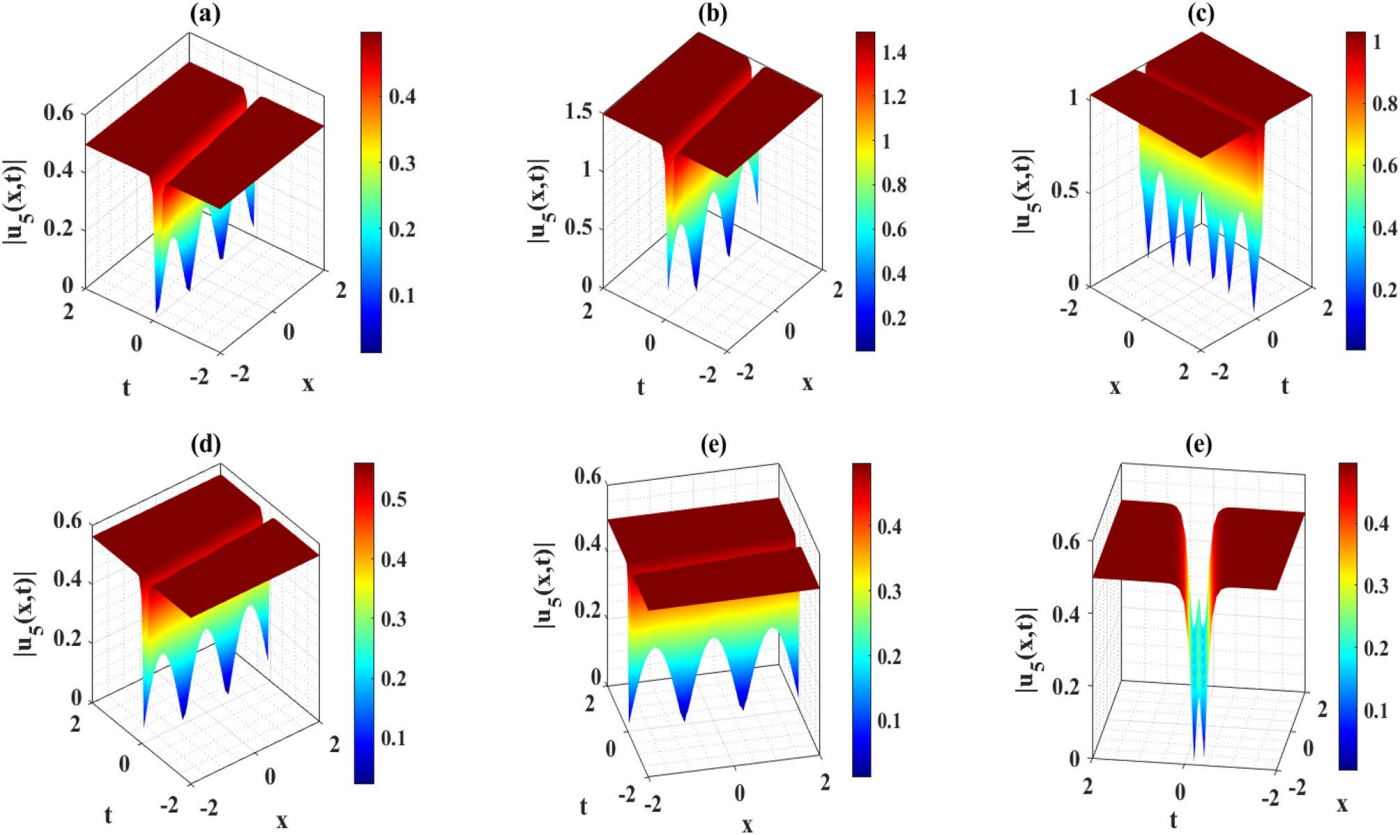
Representation of the three-dimensional wave profiles of the solution ${u}_{5}\left(x,t\right)$ for the selected parameters $Q=2, a=1,\lambda =0.1, q=5.1, \sigma =0.2$. (**a**) $\chi =0.21, \gamma =1, \mu =2,\varepsilon =0.2, \eta =0.3, \rho =0.01$; (**b**) $\chi =0.21, \gamma =5, \mu =2,\varepsilon =0.2, \eta =0.3, \rho =0.01$; (**c**) $\chi =0.21, \gamma =1, \mu =3,\varepsilon =0.2, \eta =0.3, \rho =0.01$; (**d**) $\chi =0.21, \gamma =1, \mu =2,\varepsilon =-0.1, \eta =0.3, \rho =0.01$; (**e**) $\chi =0.21, \gamma =1, \mu =2,\varepsilon =0.2, \eta =0.3, \rho =0.01$; **(f)**: $\chi =0.21, \gamma =1, \mu =2,\varepsilon =0.2, \eta =0.3, \rho =0.05$.

**Figure 2:**
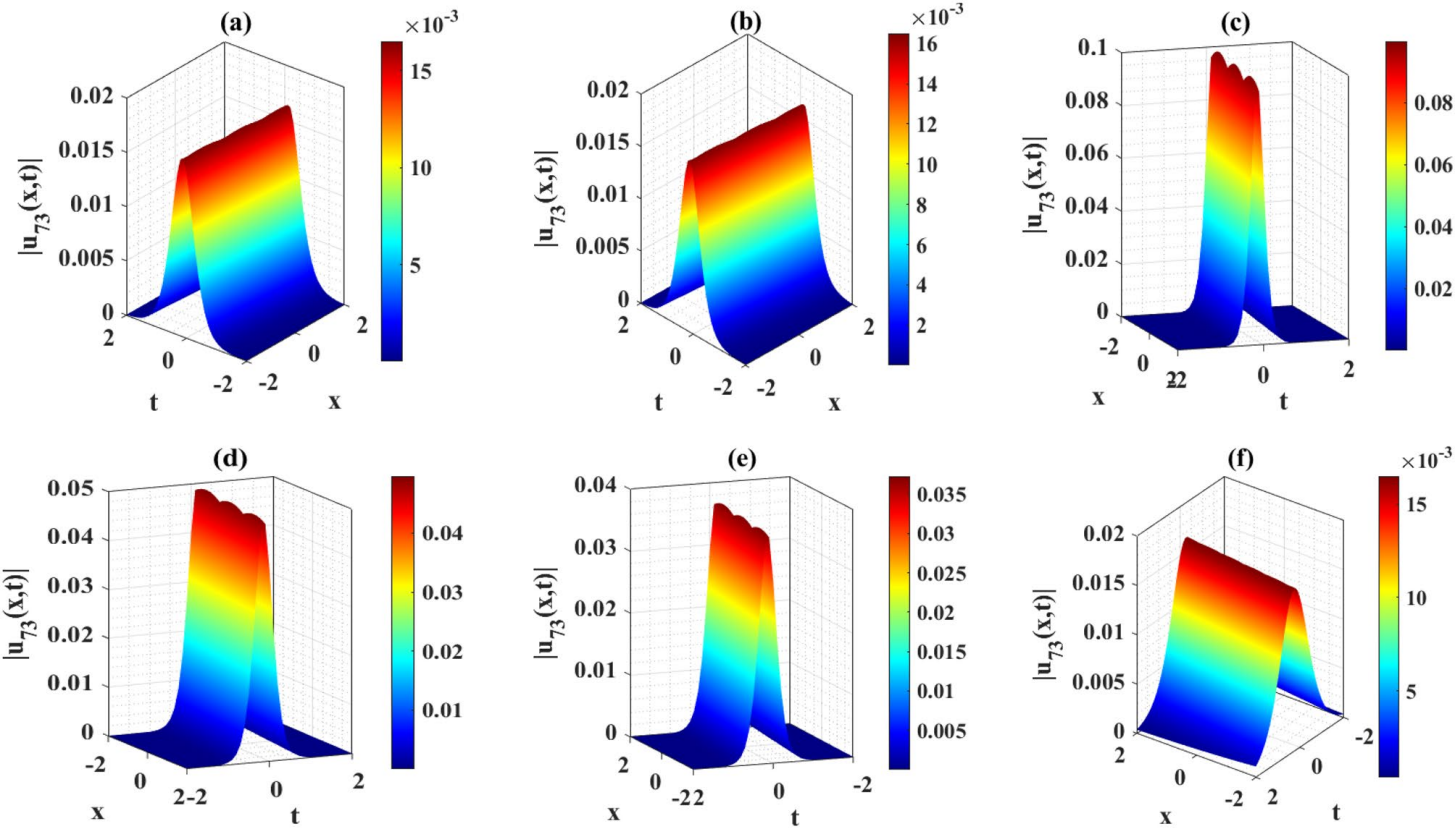
Representation of the three-dimensional wave profiles of the solution ${u}_{73}\left(x,t\right)$ for the selected parameters $Q=2, a=1,\mu =0, q=5.1, \varepsilon =0.2$. (**a**) $\chi =0.21, \gamma =1, \lambda =-0.1,\sigma =0.2, \eta =0.3, \rho =0.01$; (**b**) $\chi =0.21, \gamma =1, \lambda =-0.1,\sigma =0.2, \eta =0.3, \rho =0.01$; (**c**) $\chi =0.21, \gamma =1, \lambda =-0.6,\sigma =0.2, \eta =0.3, \rho =0.01$; (**d**) $\chi =0.21, \gamma =1, \lambda =-0.1,\sigma =0.6, \eta =0.3, \rho =0.01$; (**e**) $\chi =0.21, \gamma =1, \lambda =-0.1,\sigma =0.2, \eta =0.4, \rho =0.01$; (**f**) $\chi =0.21, \gamma =1, \lambda =-0.1,\sigma =0.2, \eta =0.3, \rho =0.03$.

A lot of parameters are involved in Eq. (1.1). Due to this, in order to illustrate the 3D wave profiles of the selected solutions, we will graphically depict the results by changing the values of the parameters related to the solutions obtained in this sub-section. It is important to note that we have constructed trigonometric, hyperbolic, exponential, and rational wave solutions to the DD model in a variety of ways. By setting numerous individual values to the derived solutions, the soliton profile for each solution is formed. The structural composition of the aforementioned solutions is characterized by the propagation of wave profiles organized alphabetically according to the $w$ type. Figure 1 illustrates the behavior of ${u}_{5}(x,t)$ as it evolves under varying parameter values and specific coefficients. On the other hand, Fig. 2 represents the bell shape wave structure of the solution ${u}_{73}(x,t)$ as it evolves under varying parameter values and specific coefficients. Bell-shaped wave profiles refer to a particular shape of waveforms or curves that resemble the form of a bell. These profiles typically exhibit a central peak or maximum point with gradually decreasing amplitudes on both sides. Bell-shaped wave profiles are fundamental in understanding various natural and scientific phenomena.

### Impact of the parameters some of the attained solutions

**Figure 3:**
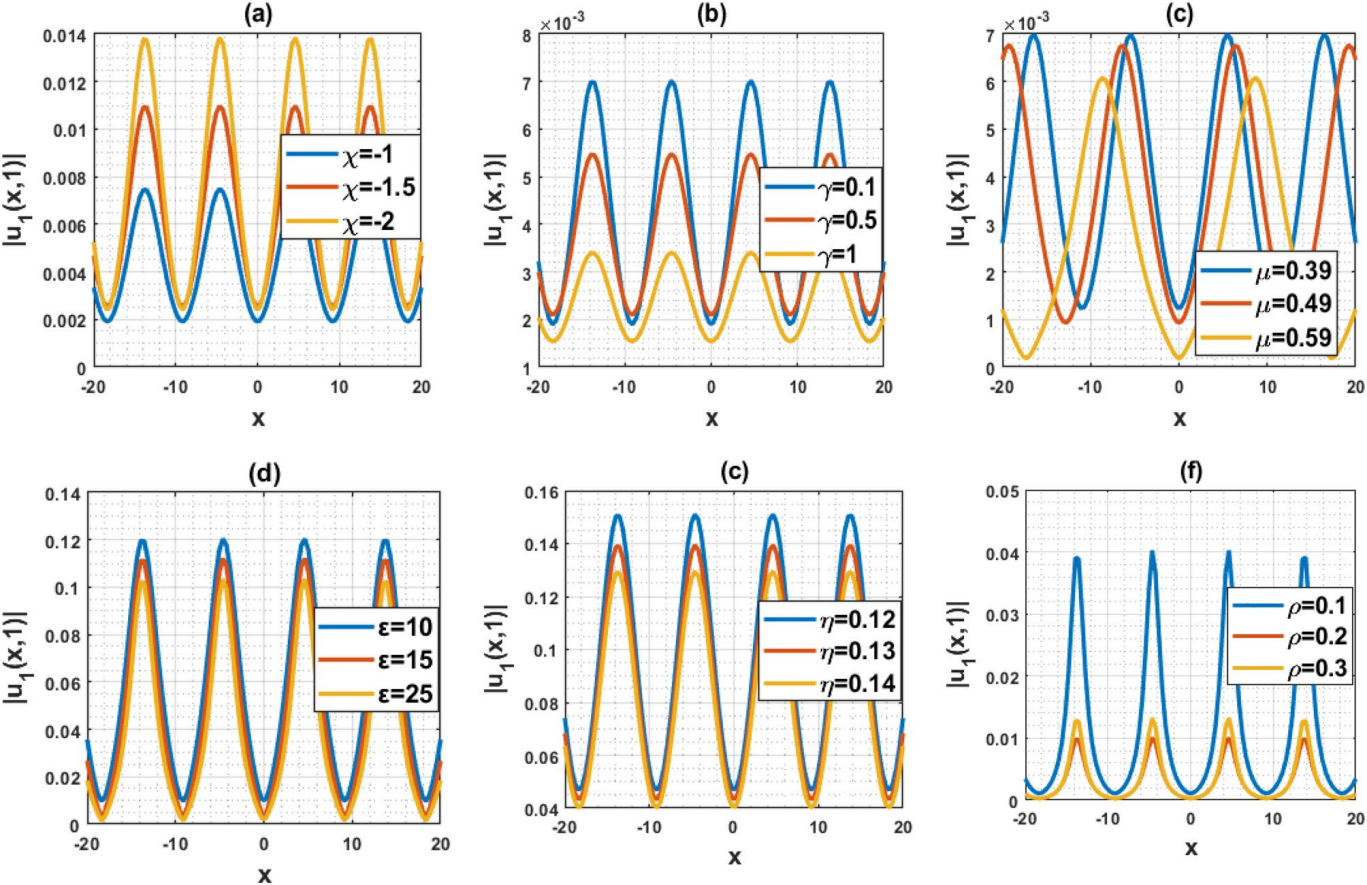
Representation of the impact of the various parameters of the solution ${u}_{1}\left(x,1\right)$.

**Figure 4:**
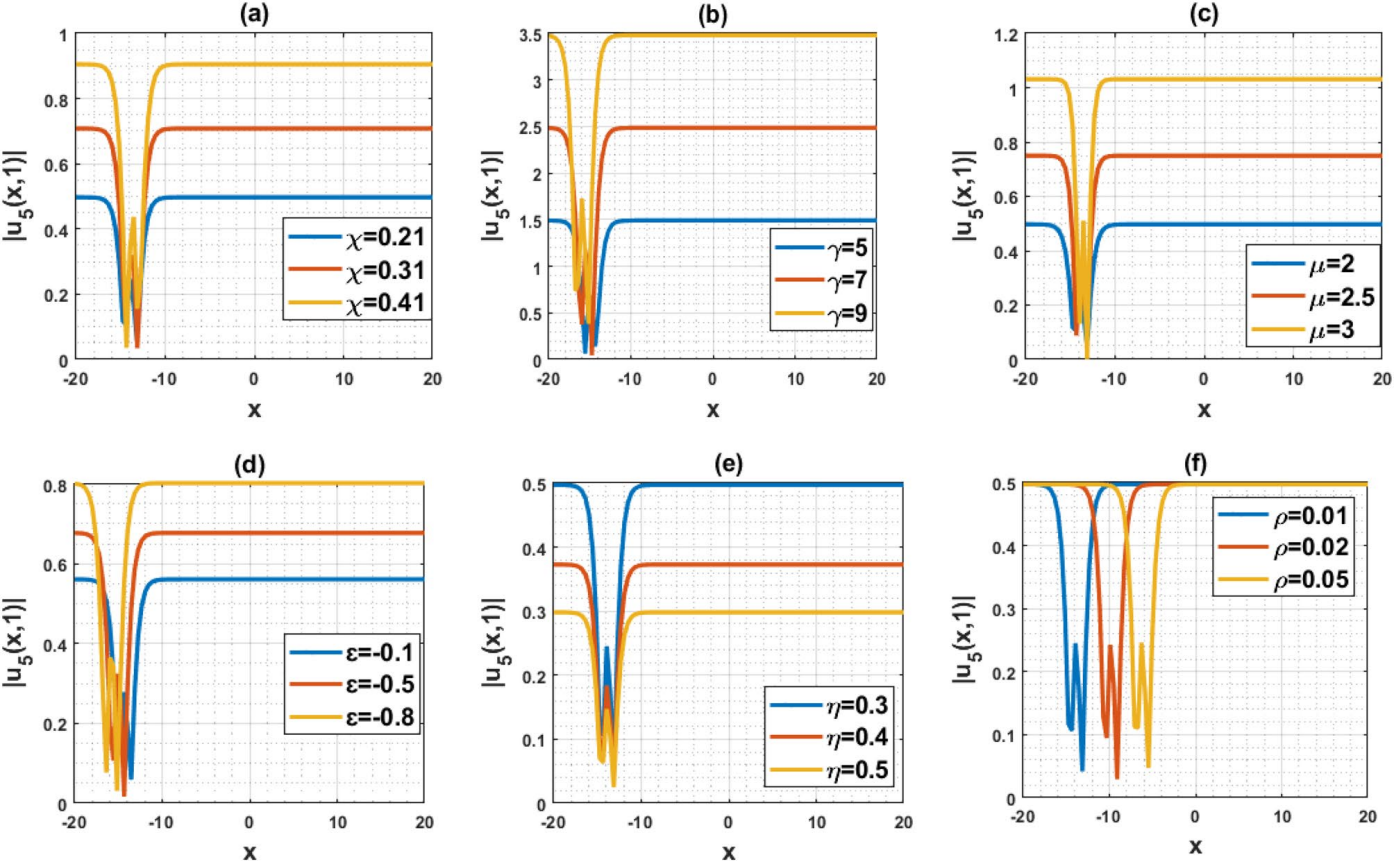
Representation of the impact of the various parameters of the solution ${u}_{5}\left(x,1\right)$.

**Figure 5:**
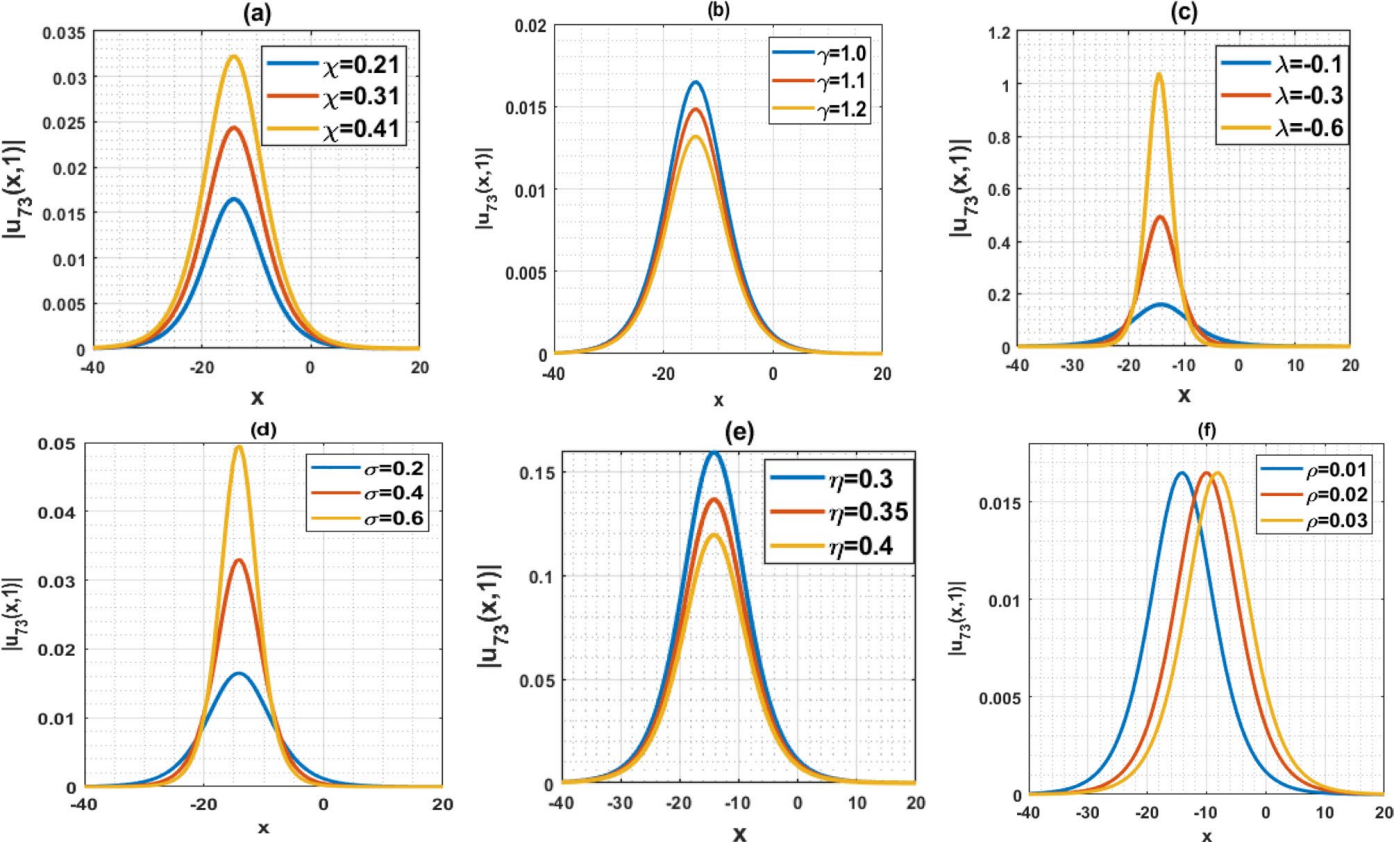
Representation of the impact of the various parameters of the solution ${u}_{73}\left(x,1\right)$.

**Figure 6:**
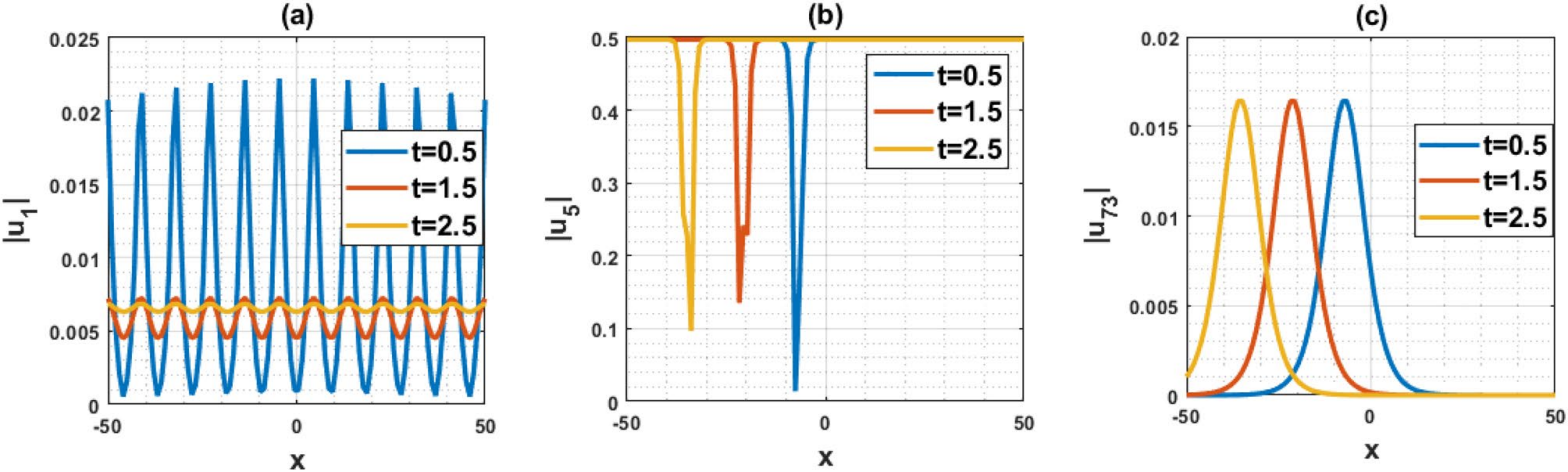
Representation of the wave profile for different time and different solutions.

The DD model constitutes a nonlinear partial differential equation characterized by a second-order temporal derivative. Its application has been directed towards examining strain wave propagation within Murnaghan’s rod, particularly concerning scenarios involving elastic inhomogeneous media. This model has been involved lot of important parameters such as $\rho$ denotes the density, $\varepsilon$ denotes the small parameters, $q=\frac{B}{Q}$ and $a=\frac{M}{Q}$ denotes the scale factors, $\gamma$ denotes the poisson parameter and others. In this paper, we have used the MK scheme on mentioned model and constructed different types of solutions for Murnaghan’s rod including lot of parameters. In this sub-section, we will discuss the impact of parameters on the stated model by using the two-dimensional combined chart. As a result, we analyze the parameter values: $\chi =-1, Q=-0.2, a=-0.1,\gamma =0.02,\mu =0.1, \lambda =0.3,\sigma =0.4, q=-5.1, \eta =0.3, \varepsilon =4, \rho =0.1$, In Fig. 3a, the behavior of the solution $\left|{{\text{u}}}_{1}\left(x,t\right)\right|$ is depicted, specifically focused on the parameter $\chi$ and its influence. This solution exhibits a periodic waveform. Furthermore, Fig. 3b–e illustrate the impact of various parameters on the solution $\left|{{\text{u}}}_{1}\left(x,t\right)\right|$ both contributing to periodic waveform profiles. These profiles highlight the occurrence of periodic wave phenomena, particularly noteworthy in the context of Murnaghan's rod. Analyzing Fig. 3b–e reveals a reduction in the amplitude of the waveform as parameters $\gamma , \mu , \varepsilon$ and $\eta$ are decreases. Conversely, Fig. 3a and f show an increase in waveform amplitude with decreasing values of parameter $\chi$ and $\rho$. For the Fig. 4, we analyze the parameter values: $\chi =0.21, Q=2, a=1,\gamma =1,\mu =2, \lambda =0.1,\sigma =0.2, q=5.1, \eta =0.3, \varepsilon =0.2, \rho =0.01$, In Fig. 4a–f, the behavior of the solution $\left|{{\text{u}}}_{5}\left(x,t\right)\right|$ is depicted, specifically focused on the various parameters $\chi$ $\gamma , \mu , \varepsilon ,\rho$ and $\eta$ and its influence. This solution exhibits an alphabetically $w$-shape waveform. These profiles highlight the occurrence of $w$-shape wave phenomena, particularly noteworthy in the context of Murnaghan's rod. Figure 5a–f represents the bell shape wave profile of the solution $\left|{{\text{u}}}_{73}\left(x,t\right)\right|$ for selecting the parameters $\chi =0.21, Q=2, a=1,\gamma =1,\mu =0, \lambda =-0.1,\sigma =0.2, q=5.1, \eta =0.3, \varepsilon =0.2, \rho =0.01$ and also displayed the influence of the various parameters $\chi$ $\gamma , \mu , \varepsilon ,\rho$ and $\eta$. This solution exhibits a bell shape waveform. These profiles are also effective the occurrence of bell shape wave phenomena, particularly noteworthy in the context of Murnaghan's rod. Analyzing from the figures, it can be seen that the nature of the wave profile changes for the particular values of the selecting parameters $\chi$ $\gamma , \mu , \varepsilon ,\rho$ and $\eta$. Finally, Fig. 6a–c represents the nature of the wave profiles of the selected solutions for different time. It can be seen that the behaviors of the wave profiles changes with changes of the time. The solutions derived from the DD model are highly valuable as they find applicability in explaining diverse physical phenomena, including but not limited to shock waves, tsunamis, and solitons.

### Bifurcation analysis of the DD model

In this study, we investigate the novel dynamics of the DD model via Eq. (1.1) using concepts from bifurcation theory. By introducing the variables $X=\phi$ and $Y={X}{\prime}$, Eq. (2.2) can be transformed into a planar dynamical system with the following form:

3.1 $$\left\{\begin{array}{c}\frac{dX}{d\xi }=\phi \\ \frac{dY}{d\xi }=-\frac{2\left(Q-{\omega }^{2}\rho \right)}{\varepsilon \chi \left({\omega }^{2}\rho -\gamma a\right)}X-\frac{q\eta \varepsilon }{\varepsilon \chi \left({\omega }^{2}\rho -\gamma a\right)}{X}^{2}\end{array}\right..$$

This phase plane representation corresponds to the familiar phase plane linked to wave solutions of the DD model. Using the Hamilton canonical equations ${X}{\prime}=\frac{\partial H}{\partial Y}$ and ${Y}{\prime}=-\frac{\partial H}{\partial X}$, the Hamiltonian function comes from the system (3.1) as

3.2 $$H\left(X,Y\right)=\frac{{Y}^{2}}{2}+\frac{Q-{\omega }^{2}\rho }{\varepsilon \chi \left({\omega }^{2}\rho -\gamma a\right)}{X}^{2}+\frac{q\eta \varepsilon }{3\varepsilon \chi \left({\omega }^{2}\rho -\gamma a\right)}{X}^{3}.$$

**Figure 7:**
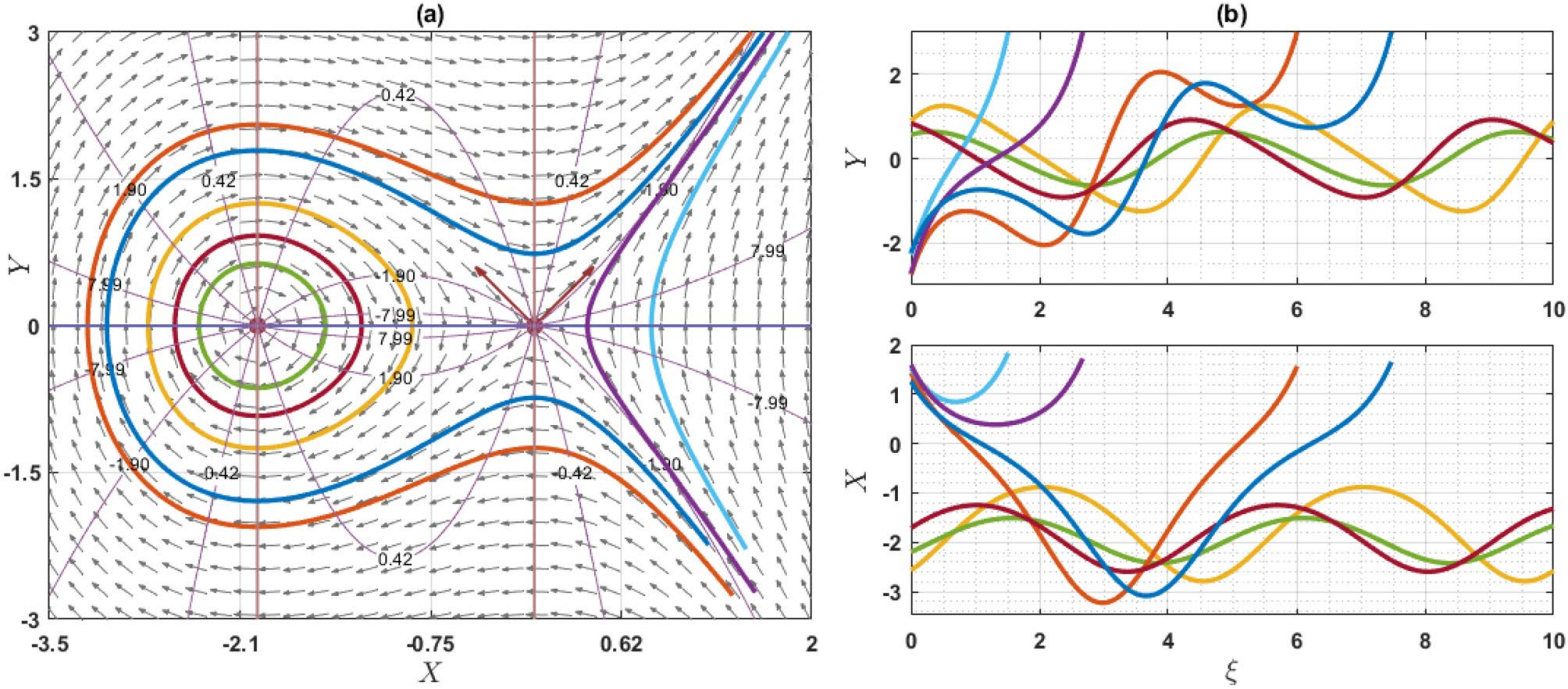
A phase profile of the Eq. (3.1) is represented as (**a**) shows its trajectories, isoclines, and nullclines, revealing its behavior. (**b**) Displays the trajectories of the consistent solutions to the wave variable $\xi$.

**Figure 8:**
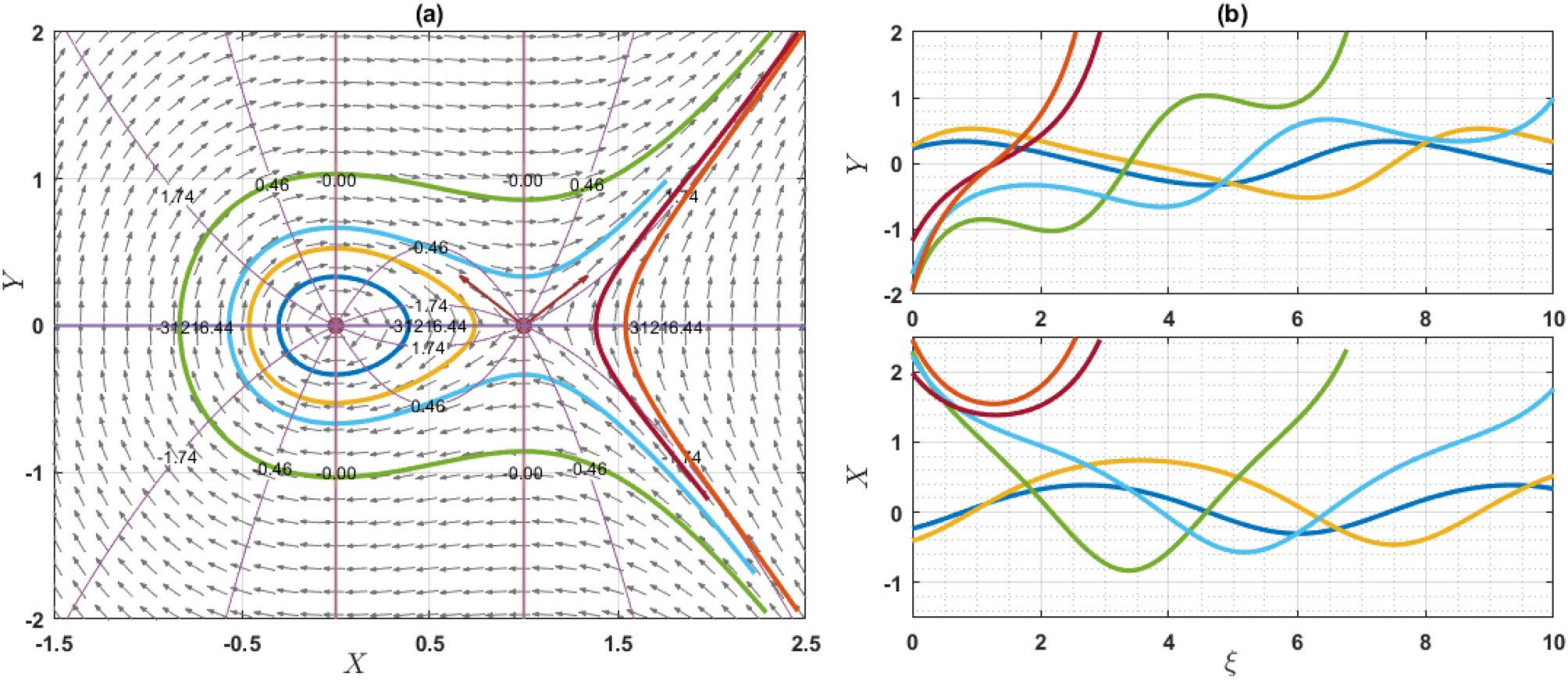
A phase profile of the Eq. (3.1) is represented as (**a**) shows its trajectories, isoclines, and nullclines, revealing its behavior. (**b**) Displays the trajectories of the consistent solutions to the wave variable $\xi$.

**Figure 9:**
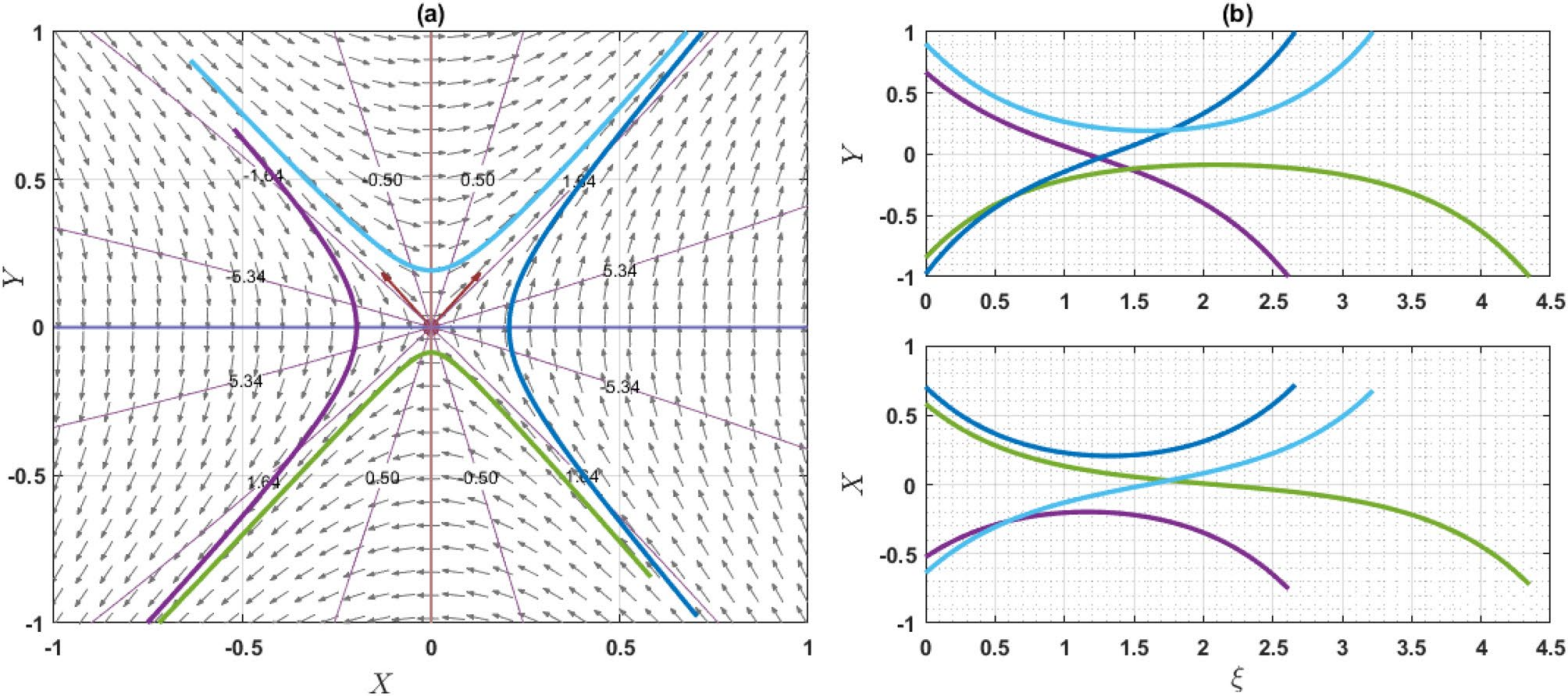
A phase profile of the Eq. (3.1) is represented as (**a**) shows its trajectories, isoclines, and nullclines, revealing its behavior. (**b**) Displays the trajectories of the consistent solutions to the wave variable $\xi$.

**Figure 10:**
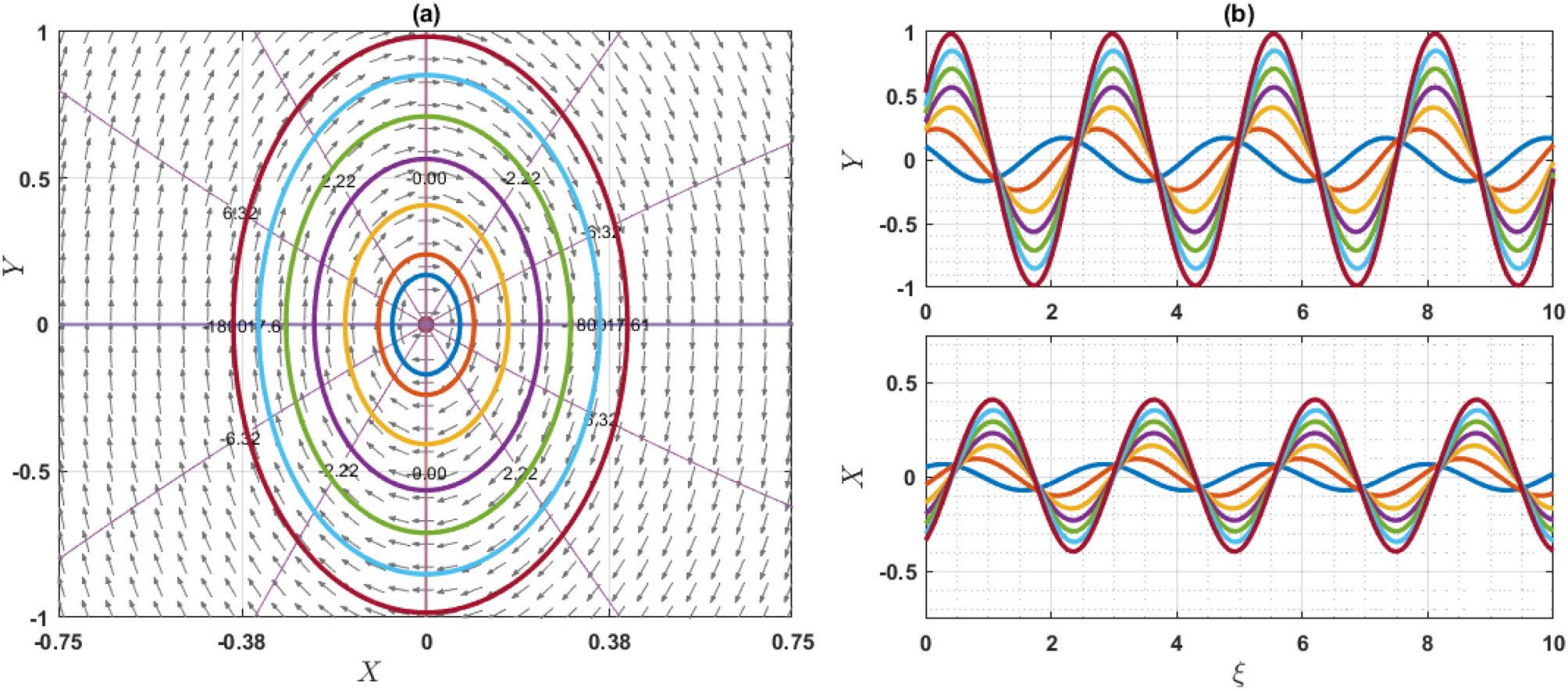
A phase profile of the Eq. (3.1) is represented as (**a**) shows its trajectories, isoclines, and nullclines, revealing its behavior. (**b**) Displays the trajectories of the consistent solutions to the wave variable $\xi$.

**Figure 11:**
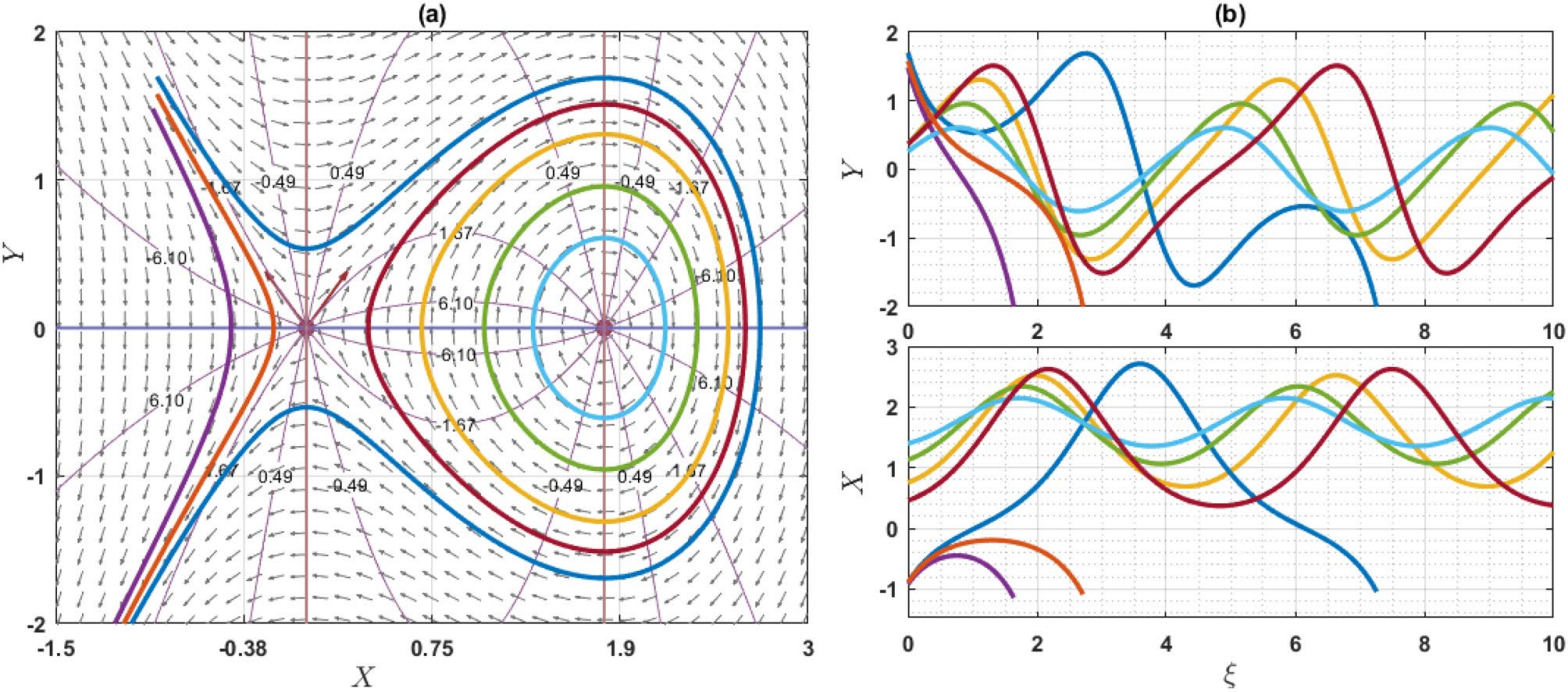
A phase profile of the Eq. (3.1) is represented as (**a**) shows its trajectories, isoclines, and nullclines, revealing its behavior. (**b**) Displays the trajectories of the consistent solutions to the wave variable $\xi$.

**Figure 12:**
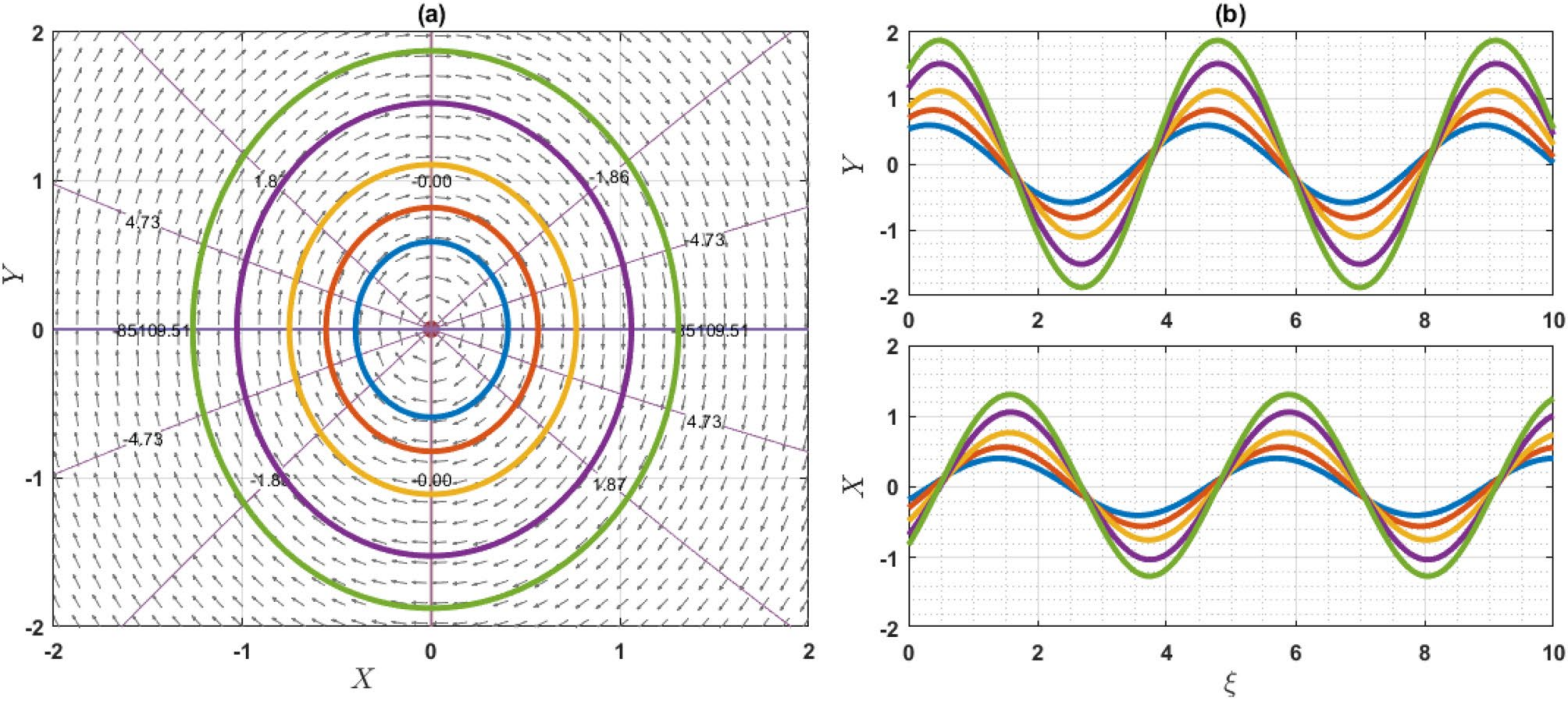
A phase profile of the Eq. (3.1) is represented as (**a**) shows its trajectories, isoclines, and nullclines, revealing its behavior. (**b**) Displays the trajectories of the consistent solutions to the wave variable $\xi$.

If $Q={\omega }^{2}\rho$, the system has only one equilibrium point (EP) as $(\mathrm{0,0})$. On the other hand, if $Q\ne {\omega }^{2}\rho$, then the system has two EP such as $\left(\mathrm{0,0}\right)$ and $\left(\frac{2\left({\omega }^{2}\rho -Q\right)}{q\eta \varepsilon },0\right)$. Note that $q\eta \varepsilon \ne 0$. For the point $\left(0, 0\right)$, the characteristics roots are $\sqrt{\frac{2\left({\omega }^{2}\rho -Q\right)}{\varepsilon \chi \left({\omega }^{2}\rho -\gamma a\right)}}$ and $-\sqrt{\frac{2\left({\omega }^{2}\rho -Q\right)}{\varepsilon \chi \left({\omega }^{2}\rho -\gamma a\right)}}$, provided that ${\omega }^{2}\rho \ne \gamma a$. If the value of $\frac{2\left({\omega }^{2}\rho -Q\right)}{\varepsilon \chi \left({\omega }^{2}\rho -\gamma a\right)}$ is grater than zero, then the eigenvalues are the real and opposite sign. So, the EP $\left(0, 0\right)$ is unstable saddle. If $\frac{2\left({\omega }^{2}\rho -Q\right)}{\varepsilon \chi \left({\omega }^{2}\rho -\gamma a\right)}<0$, then the eigenvalues are purely imaginary and the given EP is stable center or ellipse. On the other hand, for another point $\left(\frac{2\left({\omega }^{2}\rho -Q\right)}{q\eta \varepsilon },0\right)$, the characteristics roots are $\sqrt{\frac{2\left(Q-{\omega }^{2}\rho \right)}{\varepsilon \chi \left({\omega }^{2}\rho -\gamma a\right)}}$ and $-\sqrt{\frac{2\left({\omega }^{2}\rho -Q\right)}{\varepsilon \chi \left({\omega }^{2}\rho -\gamma a\right)}}$, provided that ${\omega }^{2}\rho \ne \gamma a$. If the value of $\frac{2\left({\omega }^{2}\rho -Q\right)}{\varepsilon \chi \left({\omega }^{2}\rho -\gamma a\right)}$ is grater than zero, the eigenvalues are real and opposite sign and thus the EP is unstable saddle point. If $\frac{2\left({\omega }^{2}\rho -Q\right)}{\varepsilon \chi \left({\omega }^{2}\rho -\gamma a\right)}<0$, the eigenvalues are purely imaginary. So, the EP is stable center point. For different choices of the parameters, we will explain as: For the values of the parameters $Q=2, \omega =1, q=1, \chi =1, \gamma =2, a=1,\varepsilon =1, \rho =1$ and $\eta =1$ as display the phase portrait of the model, which is shown in Fig. 7. We have been seen that the two EPs where $(\mathrm{0,0})$ is unstable saddle and $(-2, 0)$ is center points. Figure 8 exhibits the phase portrait of the values of the parameters $Q=0.5, \omega =1, q=1, \chi =1, \gamma =2, a=1,\varepsilon =1, \rho =1$ and $\eta =1$. We have been seen that the two EPs where $(\mathrm{0,0})$ is center and $(1, 0)$ is unstable saddle points. Figure 9 displays the phase portrait of the values of the parameters $Q=1, \omega =0.1, q=1, \chi =1, \gamma =1, a=1,\varepsilon =1, \rho =1$ and $\eta =0.2$. We have been seen that only one EP as $(\mathrm{0,0})$ is unstable saddle point. Figure 10 presents the phase portrait of the values of the fixed parameters $Q=-2, \omega =1, q=1, \chi =1, \gamma =2, a=1,\varepsilon =1, \rho =1$ and $\eta =1$. We have been seen that only one EP as $(\mathrm{0,0})$ is center. Figure 11 presents the phase portrait of the values of the fixed parameters $Q=2, \omega =1.7, q=0.5, \chi =1, \gamma =0.1, a=1.5,\varepsilon =1, \rho =1$ and $\eta =1$. We have been seen that two EPs where $(\mathrm{0,0})$ is unstable center and $\left(1.78, 0\right)$ is center. If the change of the values of the parameters $\chi$, then the remain unchanged figures like as center and unstable saddle points. For the values of the parameters $Q=0.5, \omega =1.7, q=0.1, \chi =-1, \gamma =-2, a=-1,\varepsilon =1, \rho =1$ and $\eta =1$, Fig. 12 display the phase portrait of the model. We have been seen that only one EP as $(\mathrm{0,0})$ is center point. Due to this discussion, it can be seen that the presence of nonlinear periodic trajectory and nonlinear homoclinic trajectory ensure the occurrence of the solutions of the DD model. It is also seen that the wave solutions of the trajectories are not obtained. In summary, phase plane analysis is a valuable technique that plays a fundamental role in understanding the dynamics, stability, and behavior of complex systems in various scientific and engineering disciplines. It provides a visual and intuitive way to interpret and predict the evolution of systems, making it an indispensable tool for researchers and engineers alike.

## Conclusion

This article presents a study on the DD model, where various wave solutions are derived through the MK method. These solutions encompass both fresh and classical soliton patterns known in existing literature. The wave solutions are associated with several free parameters related to the approach and the model, and their effects are discussed. We have plotted the 3D diagrams some of the selected solutions as shown in Figs. 1 and 2. Furthermore, 2D combined diagrams are illustrated to examine the impact of these parameters at different levels as shown in Figs. 3, 4, and 5. To understand the dynamical behaviors of solitons in various disciplines, stability analysis is performed for some of the obtained solutions. Additionally, bifurcation analysis of the model is carried out. The stability of equilibrium points is analyzed, and the phase portrait of the system are depicted in Figs. 7, 8, 9, 10, 11, and 12. The results suggest that changes in parameter values can lead to shifts in the dynamics of wave solutions provided by the DD model. Overall, the MK scheme proves to be a powerful, compatible, and straightforward method to derive comprehensive wave solutions with various free parameters, which are valuable for describing wave profiles in different scenarios. Therefore, it is reliable, easy to use and effective, future research can use the implemented method to obtain analytical wave solutions to incremental nonlinear fractional wave equations of many models in nonlinear science and engineering.

## Data Availability

The authors confirm that the data supporting the findings of this study are available within the article.
